# A general linear model-based approach for inferring selection to climate

**DOI:** 10.1186/1471-2156-14-87

**Published:** 2013-09-22

**Authors:** Srilakshmi M Raj, Luca Pagani, Irene Gallego Romero, Toomas Kivisild, William Amos

**Affiliations:** 1 Department of Biological Anthropology, Henry Wellcome Building, University of Cambridge, Fitzwilliam Street, Cambridge, UK; 2 Present address: Department of Molecular Biology and Genetics, Cornell University, Ithaca, NY, USA; 3 Department of Human Genetics, University of Chicago, Chicago, IL, USA; 4 Department of Zoology, University of Cambridge, Cambridge, UK

**Keywords:** Climate, Adaptation, Human evolution, Natural selection, Environmental adaptation, Population genetics

## Abstract

**Background:**

Many efforts have been made to detect signatures of positive selection in the human genome, especially those associated with expansion from Africa and subsequent colonization of all other continents. However, most approaches have not directly probed the relationship between the environment and patterns of variation among humans. We have designed a method to identify regions of the genome under selection based on Mantel tests conducted within a general linear model framework, which we call MAntel-GLM to Infer Clinal Selection (MAGICS). MAGICS explicitly incorporates population-specific and genome-wide patterns of background variation as well as information from environmental values to provide an improved picture of selection and its underlying causes in human populations.

**Results:**

Our results significantly overlap with those obtained by other published methodologies, but MAGICS has several advantages. These include improvements that: limit false positives by reducing the number of independent tests conducted and by correcting for geographic distance, which we found to be a major contributor to selection signals; yield absolute rather than relative estimates of significance; identify specific geographic regions linked most strongly to particular signals of selection; and detect recent balancing as well as directional selection.

**Conclusions:**

We find evidence of selection associated with climate (P < 10^-5^) in 354 genes, and among these observe a highly significant enrichment for directional positive selection. Two of our strongest 'hits’, however, *ADRA2A* and *ADRA2C*, implicated in vasoconstriction in response to cold and pain stimuli, show evidence of balancing selection. Our results clearly demonstrate evidence of climate-related signals of directional and balancing selection.

## Background

Within the last 100,000 years humans dispersed from Africa to occupy most of the habitable space in the world. During this process our species has successfully combined cultural buffering, biological plasticity and adaptation to cope with the wide range of new ecosystems, pathogens and climates they encountered [1-3]. Climate, in particular, comprises many diverse elements such as temperature, humidity, precipitation and solar radiation, so it would be surprising if many different genes had not been influenced by natural selection. Indeed, many physiological traits exhibit geographic trends that correlate with climate [4-8]. However, without an explicit link to global patterns of genetic variation, the extent to which these trends reflect adaptation through natural selection remains unclear.

Many genetic studies on humans have attempted to identify genes and genomic regions associated with regional adaptation by looking for signatures of selection [2,9-15]. These studies have relied on a diverse range of approaches that mostly identify outliers in the empirical genome-wide data, including searches for markers exhibiting unusually high levels of geographic differentiation [2,9], for genomic regions with high linkage disequilibrium and derived allele frequency [10], and for markers where the loss of genetic variability that occurred when humans migrated out of Africa has been particularly high or low [11-14]. These approaches suggest that a substantial proportion of the human genome contains candidates of positive selection [15]. However, it can be difficult to ascribe environmental or biological factors to any particular signal. Furthermore, wherever signatures of selection are sought by considering patterns of genetic variation in isolation, i.e. without reference to a specific hypothesis, it can become difficult to separate genuine signals from those that arise from other sources including genotyping errors and other artifacts.

One way to increase statistical power when searching for signatures of selection is to study patterns of genomic variation across populations in relation to particular environmental characteristics. For example, physiological adaptations to temperature and solar radiation, as well as several other traits, have been shown to vary along a latitudinal cline [16-18], suggesting selection by climate. Even modest regional allele frequency differences can provide evidence of selection if they correlate strongly with one or more environmental variables, provided the environmental variables are accurately measured and also approximate the selective pressure over the time of evolution. Explored earlier by Prugnolle et al. (2005) [19], this approach has been pioneered by Hancock et al. [20-23], who use a Bayesian algorithm [24] to search for markers at which variations in allele frequency correlate more than the genomic average with global variation in one or more climatic variables. In this approach, absolute significance is not determined. Instead, markers are ranked in terms of their degree of association. On the one hand this makes the approach sensibly conservative, but on the other it precludes a meaningful estimate of the proportion of the genome actually influenced by selection.

Here we present a new approach for detecting signatures of selection based on the use of general linear models to analyze similarity matrices. This framework allows three important advantages. First, data from neighboring markers can be combined into a single genetic window, thereby reducing greatly the number of independent tests that need to be performed. Second, the method is flexible, allowing incorporation of possible cofactors such as geographic distance between populations and interactions between variables. In particular, by fitting genome-wide genetic relatedness we can control for variation in the level of shared ancestry between different pairs of individuals or populations. Third, statistical significance is determined through a form of Mantel test, based on repeated randomization (scrambling) of the data at one predictor variable, allowing absolute estimates of significance rather than empirical ranking. We apply this approach to genome-wide data from 45 global populations using four climate variables, each quantified in both the summer and the winter seasons. We then compare our results with those of Hancock et al. (2011) [21] available through the dbCLINE online database (http://genapps2.uchicago.edu:8081/dbcline/main.jsp), and identify a number of overlapping genomic regions as candidates for recent selection.

## Results

The method we propose is designed to identify regions of the genome that have experienced climate-related selection since modern humans colonized the world 'out of Africa’. We aim simultaneously to reduce the impact of false positives, by radically reducing the necessary number of independent tests, and to minimize the impact of observations that are unusual for reasons other than natural selection, for example genotyping errors. MAGICS is based on the identification of genomic regions where genetic similarity between populations correlates with climatic similarity, after correcting for factors such as genome-wide relatedness and geographic distance.

Previous analyses of genome-wide data and climate have tended to use a 'linear’ framework in the sense that there is a 1:1 correspondence between a given marker and the trait being measured. For example, one might conduct a regression of solar radiation index against allele frequency. With MAGICS, all variables are transformed into similarity or distance matrices. Thus, solar radiation index is scored as pairwise differences in solar radiation index between geographic regions, while genotype data are scored variously as genotype identity (one locus, same or different), relatedness (between individuals over multiple loci) or genetic distance (between populations, multiple loci). The extent to which two or more similarity matrices are correlated is classically determined using a Mantel test, in which the raw correlation between linearized matrices is tested by repeated randomization. We extend this slightly by fitting general linear models instead of simple correlations or multiple regressions. This allows for the inclusion of factors as well as continuous variables and, where desired, for inclusion of interaction terms between variables. As in a classical Mantel test, significance is determined by randomization of one predictor variable.

With this approach, and given the climatic and genetic data from a range of globally distributed populations, we seek to fit models of the form:

$${\mathrm{LocalF}}_{\mathrm{ST}}\;\sim \;{\mathrm{GWF}}_{\mathrm{ST}}+\mathrm{Geography}+\mathrm{Climate}$$

where the response variable, LocalF_ST_, is the genetic relatedness between populations/individuals at a given genomic location, and the predictor variables are: GWF_ST_ or genome wide F_ST_, defined as the genetic distance between pairs of populations based on all available SNPs across the genome; Geography, defined as the land-only distance between population pairs [25]; and Climate, the difference between pairs of populations at a climate measurement of interest. In this way we ask the extent to which a given genomic region differs more or less than expected among populations, relative to variation in the entire rest of the genome, and at the same time determine whether this measure of difference covaries systematically with the climatic difference between the regions. Geographic distance is included as a conservative factor to control for deviations from a simple isolation by distance model. This might include instances where genetically dissimilar populations live in close proximity or where migration has caused genetically very similar populations to be physically distant. Our reasoning is that selective forces due to disease, for example, may differ greatly between regions with similar climate on different continents. Local F_ST_ was calculated across 'genic windows’ comprising all SNPs located ± 25Kb from the midpoint of each gene; this 50Kb window size encompasses the full transcript of roughly 65% of genes in the human genome [26]. Using 'genic windows’ enabled us to focus on regions of the genome annotated by function, compare our results to those from similar studies and account for variability in gene size. Alternative approaches based on fixed or sliding windows could also be used but were not pursued in the current study. No attempt was made to weight SNPs by their function in coding, promoter or enhancer regions, nor to exclude SNPs lying outside shorter genes, although both of these are possible improvements that would be worth exploring in future work.

We applied the MAGICS approach to 28,784 genic windows for which SNP data were available in a genome-wide data set of 45 human populations. Given known issues with ascertainment bias [27,28] affecting particularly African – non-African population comparisons, most African populations were omitted, the exception being Egypt where geographic and genetic distance fits well with the pattern of isolation by distance observed outside Africa. We searched for signatures of selection associated with four climate variables over two seasons, summer and winter. To explore the impact of different data scrambling strategies we repeated the entire analysis twice. In the first run the climate variable was scrambled across the entire dataset, as in a classical Mantel test (CS = 0). In the second run we employed a more conservative approach whereby scrambling was restricted to within each of six continents (CS = 1). The latter will tend to exclude association driven entirely by differences between continents. Over both analyses we identified a total of 397 significant associations (P < =10^-5^,’hits’) spread across 354 unique genes included in as many genic windows. Since some hits involve multiple, adjacent overlapping genic windows and are therefore non-independent, we also estimated the likely number of independent hits. For this we define 'genic region’ as contiguous groups of genic windows where no P value > 10^-3^. Our hits represent 1.2% of the genic windows analyzed, and map to 317 different genic regions (Table 1). The full list of hits by climate variables and associated Ensembl Gene IDs are provided in Additional file 1: Table S3. Approximately 93% of these associations (328 out of 397 hits) were revealed using the global scrambling (CS = 0) strategy. Adding the continent-specific scrambling option (CS = 1) was, as expected, more conservative, yielding only 69 hits. The overlap between CS = 0 and CS = 1 was small but significantly non-random (Table 1, chi-square, P = 1.2 × 10^-64^).

**Table 1:** Count of hits for each climate variable

**a. Summary of hits**				
	**Solar radiation**	**Relative humidity**	**Temperature**	**Precipitation**
**CS = 0**				
**Summer**	7	55	4	59
**Winter**	79	5	67	52
**CS = 1**				
**Summer**	3	1	3	14
**Winter**	27	1	19	1
**CS = 0 & CS = 1**				
**Summer**	0	0	0	5
**Winter**	8	0	3	0
				
				
**b. CS = 0 and CS = 1 overlaps**				
	CS = 1	<=0.001	<=0.0001	<=0.00001
CS = 0	TOTAL	1283	235	69
<=0.001	3031	634 (135)	153 (25)	53 (7)
<=0.0001	783	269 (35)	75 (6)	23 (2)
<=0.00001	328	147 (15)	44 (3)	16 (1)

**Table 1a.** Count of hits (-log10P > =5) for each climate variable partitioned by whether significance testing was conducted by scrambling globally across all populations (CS = 0) or with scrambling restricted to within each continent (CS = 1). **Table 1b.** Dependence of number of hits on the method of randomization used in significance testing. TOTAL gives the number of genic windows that achieve the specified significance, determined using CS=0 (worldwide scrambling) or CS=1 (scrambling within continents). Numbers in the table indicate overlap between the methods with the expected overlap in brackets, calculated assuming complete independence. Thus, the bottom left cell indicates that 147 of 328 hits achieving top significance with CS=0 achieved at least the lowest level of significance with CS=1 when the expected number was 15. Of these 147, 44 achieved at least P<=0.0001, including 16 that achieved top significance.

**Figure 1:**
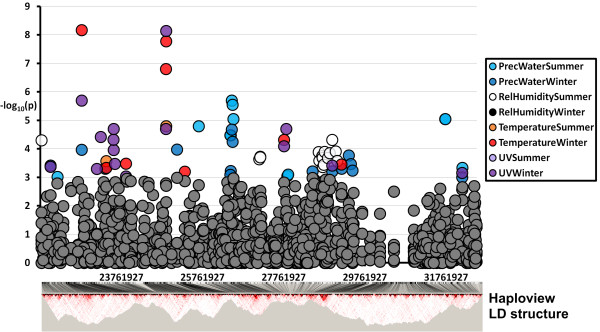
**Typical MAGICS output for a 25 Mb region on chromosome 1.** A typical MAGICS output is exemplified by a randomly selected region of chromosome 1 in which all genic windows are represented by their –log_10_P values. Windows yielding a –log_10_P > =3 are colored according to the climate variable associated with the p value. The bottom part of the plot reports the average worldwide linkage disequilibrium structure for the same region, obtained by Haploview software [29] on all the samples in the study. The LD structure is provided for the sample region to indicate the independence of MAGICS hits.

A typical output from MAGICS is presented in Figure 1 for an arbitrarily selected region spanning 25 Mb of chromosome 1. This figure illustrates several important characteristics. First, as is well-documented, genes are patchily distributed, with dense clusters interspersed by gene-poor regions, for example at ~30 Mb. The consequent clustering of hits is then translated by MAGICS into 'genic regions’. A clear example of such local clustering is the group of summer humidity associations at ~29 Mb, in a region extending more than 2 Mb. This might indicate either the existence of several genes related by their function that lie in the same region. Alternatively, this clustering could be due to locally high levels of linkage disequilibrium, as suggested in the lower panel.

The difference between using CS = 0 and CS = 1 is less than one would expect by chance. If selection occurs only on one continent, worldwide scrambling (CS = 0) will produce a far lower P-value than scrambling within continents. In contrast, if selection acts globally on the same trait and in the same way, the difference between the two scrambling regimes should be much less. Summer relative humidity yielded 55 hits with CS = 0 but only 1 with CS = 1, suggestive of localized selection, whereas winter solar radiation and winter temperature yield similar numbers of hits under both regimes and these are themselves strongly correlated on a global scale (Spearman rank ρ = 0.76). Similarly, winter precipitation and winter temperature are strongly correlated (Spearman rank ρ = 0.64) and yield similar numbers of hits for CS = 0 (Table 1, Additional file 1: Table S2). These pairs of environmental variables may therefore exert similar selection pressures and hence create parallel associations. Interestingly, this does not seem to be the case because among all our hits only 36 are associated with more than one environmental variable at a high significance level (P ≤ 10^-5^) (Additional file 1: Figure S1). Note that some lack of concordance can be attributed to a threshold effect. Where the true P-values of two climate variables are both 10^-5^, the stochasticity of randomizations will cause random variation about this expectation and in only 25% of such cases will both climate variables be judged 'hits’. This effect will operate, albeit to a lesser extent, wherever both climate variables yield similar P-values close to the threshold.

**Figure 2:**
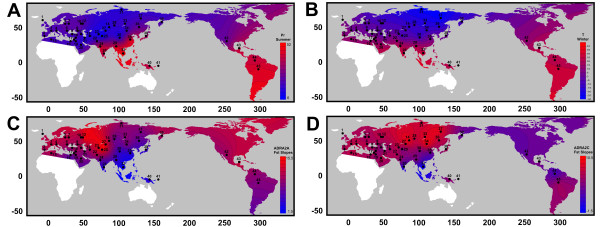
**Relationship between climate and genetic variation around genes *ADRA2A *and *ADRA2C*.** Interpolated worldwide distributions of Summer Precipitation and Winter Temperature are presented in panels **A** and **B** respectively. Our two top hits, *ADRA2A* and *ADRA2C* were identified as loci in which two or more populations exhibit unusually strong or weak genetic differentiation relative to that expect based on genome-wide F_ST_. To capture this, for each population we calculated the slope of the relationship between F_ST_ at the locus of interest and geographic separation after correcting for genomewide F_ST_. Panels **C** and **D** present heat maps of these slopes for *ADRA2A* and *ADRA2C* respectively. As seen, the climate heat maps are a close match for the inverse of the genetic differentiation heat maps.

For the 397 'genic windows’ which yielded at least one P = 0 after 10^5^ randomizations, suggestive of P values below 10^-5^, additional randomizations were added up to a maximum of 2x10^6^ to identify stronger hits. A total of 56 'genic windows’ remain with P = 0 even after the additional randomizations. We therefore implemented a data-splitting approach to facilitate P-value extrapolation, dividing the data into two randomly selected halves and then combining the two resulting P-values (see Methods). Plotting –log(P) for the full data against the equivalent value obtained by data-splitting reveals a strong linear relationship, though with appreciable scatter (Additional file 1: Figure S2). Averaging within bins exposes consistent linearity over the entire range of values explored, with a slope of 2 and intercept of -0.93. Importantly, a full-dataset P-value of 1 × 10^-6^ requires only 1000 split data randomizations, while one successful randomization in 10^6^ in each half implies an overall P-value of 10^-12^. While acknowledging that there is considerable scatter, we used this approach to assign extrapolated P-values to all 56 hits that still yielded P = 0 in 2,000,000 randomizations using the following -log_10_P_true_ = 2 (-log P_halves_) – 0.93. The two top extrapolated values are for *ADRA2A* and *ADRA2C*, two of the three subunits of the Alpha-2 Adrenergic Receptor. It is remarkable and provides strong evidence of selection that these belong to the same gene family and yet are located on different chromosomes and yield highly significant climate associations for two different variables (summer precipitation and winter temperature respectively). Furthermore, worldwide patterns of the slope of the correlation between F_ST_ and geographic distance for each of the *ADRA2A* and *ADRA2C* loci show a clear inverse relationship with both summer precipitation and winter temperature, respectively (Figure 2).

To interpret the mode of selection acting at any particular 'genic window/region’, we used the approach of Amos and Bryant [30]. Neutral variability declines linearly with land-only distance from Africa [25,31-35]. Balancing and directional selection tend respectively to retard and to accelerate loss of diversity, creating slopes that are shallower and steeper, respectively. We therefore calculated the slope of the relationship between heterozygosity and distances from Africa for all genic windows and asked whether our hits exhibit an excess of extreme slopes. An excess of extreme slopes should give the hits a higher variance, and indeed, the slopes obtained for our hit loci do exhibit a significantly higher variance than the slopes of the non-hit loci (F-test, F_353,28783_ = 1.24, P = 0.0017). We find that our hits show a significant enrichment of extreme negative slopes, indicative of directional selection. Interestingly, our top two hits both show evidence of balancing selection, as indicated by shallower than expected slopes (Additional file 1: Figure S3).

To assess the performance of MAGICS in a wider context, we compared our results with those published by Hancock et al. (2011) [21]. Since they analyze each SNP independently while we use genic windows, we extracted the most significant SNPs from their dataset in each of the 27,096 genic windows covered by both studies. For every climate variable, we explored the overlap between our top 5% of windows with the top 5% of Hancock et al. (2011)’s results and assessed the extent to which windows achieved equal ranking in both analyses. Our results are summarized in Tables 2 and 3. We find significant overlap (tested using chi-squared, all p <10^-5^) for seven out of the eight climate variables, the exception being summer solar radiation (Table 3), though it should be remembered that only P-values that comfortably exceed the 'hit’ threshold are expected to show good agreement (see above).

**Table 2:** **Overlap between our hits, defined as genic windows that yielded a significance of P < =10**^**-5 **^**with MAGICS and were also significant in the analysis of Hancock et al. (2011)**[21]**at P < = 10**^**-3**^

**Gene window number**	**Chr**	**Midpoint**	**-log**_**10**_**(p)**	**Ensembl_gene**	**Hugo_gene**	**Climate variable**
3957	1	246259095.5	5.522878745	ENSG00000200085	ENSG00000200085	T Winter
4409	2	42793437.5	5.045757491	ENSG00000200550	ENSG00000200550	Sr Winter
5939	2	204522128.5	4.769551079	ENSG00000163600	*ICOS*	T Winter
7408	3	126349236.5	4.619788758	ENSG00000221955	*SLC12A8*	Sr Winter
7818	3	173051823	8.107645718	ENSG00000186329	*TMEM212*	rH Summer
12431	6	108664283.5	6	ENSG00000112335	*SNX3*	rH Winter
13542	7	42105104.5	5.698970004	ENSG00000106571	*GLI3*	T Winter
19172	10	98101022.5	4.657577319	ENSG00000197430	*OPALIN*	Pr Summer
19390	10	112828735.5	10.1 and 5.4	ENSG00000150594	*ADRA2A*	Pr Summer and Sr Winter
20844	11	70830115	6	ENSG00000172893	*DHCR7*	Sr Winter
20915	11	74006345	8 and 5	ENSG00000077514	*POLD3*	Sr Winter and T Winter
20917	11	74074065	5.20E + 00	ENSG00000223202	ENSG00000223202	T Winter
25106	14	103201459	5.00E + 00	ENSG00000126214	*KLC1*	Sr Winter
32676	20	60900313	6.10E + 00	ENSG00000101189	C20orf20	Pr Summer

Climate variables are abbreviated as following: Pr = precipitation, rH = relative humidity, T = temperature, and Sr = solar radiation.

**Table 3:** **Numerical overlap between the hits obtained by MAGICS in our study and hits obtained by Hancock et al. (2011)**[21]**at several significance thresholds**

**Climate**	**Hancock and this study top 5% overlap**	**P of overlap**	**Our hits (P < =10**^**-5**^**)**	**Overlap between our hits and Hancock’s equivalent rank**	**This study P < =10**^**-3**^	**Hancock < =10**^**-3**^	**Overlap < =10**^**-3**^
Sr Winter	150	1.6E-23	76	2	591	215	29
Sr Summer	61	4.1E-01	6	0	94	230	0
Pr Winter	90	6.8E-03	50	0	631	210	7
Pr Summer	104	1.1E-05	53	1	460	208	10
rH Winter	91	4.7E-03	4	0	65	226	4
rH Summer	85	3.6E-02	49	0	471	226	4
T Winter	119	4.7E-10	62	2	504	198	21
T Summer	109	5.4E-07	4	0	53	229	0

Significance of overlap is obtained with a simple chi-squared test assuming a null hypothesis of complete independence.

With respect to specific genes, overlaps between the top MAGICS hits (P < = 10^-5^) and Hancock et al. hits (P <10^-3^) are summarized in Table 2. Of note are the appearances of: a) *POLD3* (OMIM: 611415) for both winter solar radiation and temperature; b) the psoriasis associated [36] solute carrier transporter locus *SLC12A8* (OMIM: 611316) for winter solar radiation; c) delta-7-sterol reductase gene *DHCR7* (OMIM: 602858), the penultimate enzyme of cholesterol synthesis, for winter solar radiation; and d) *ADRA2A* for both summer precipitation and winter solar radiation. The association between genes involved in metabolism and climate variables describing the degree of cold experienced is consistent with known associations between cold adaptations and metabolism [37].

**Figure 3:**
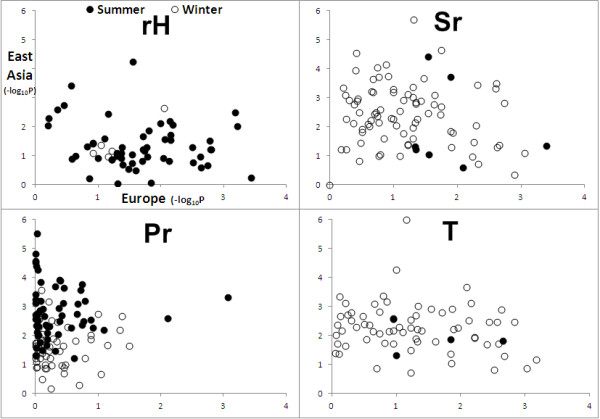
**Comparison between Europe and East Asia specific signals of selection.** At each putative hit, we further explored the origin of the signal by analyzing each continent in turn while holding all other data constant. Only the two best-represented continents, Europe and East Asia, yield meaningful signals. Panels depict –log_10_P in Europe plotted against –log_10_P for East Asia under the CS = 0 for each of the four climate variables (Pr = precipitation, rH = relative humidity, T = temperature, and Sr = solar radiation). The lack of strong correlation suggests a tendency for climate-based selection to have operated independently and largely on different subsets of genes in the different geographic regions.

To understand more about global differences in the way selection has acted we carried out further randomizations. For every hit, each continent, defined as the geographic regions in Additional file 1: Table S1, was re-analyzed separately, scrambling data only within that continent while holding all other data constant (Additional file 1: Table S3). This yielded separate P-values for each continent-genic window-climate variable combination. East Asia and Europe yielded the largest and second largest number of selection signals respectively (317 signals in East Asia, 141 in Europe), largely reflecting the fact that these two continents are represented by large numbers of populations spread over large, climatically diverse regions. Focusing on these two continents, it is clear that certain elements of climatic selection have been stronger in one compared with the other. Thus, summer and winter precipitation both reveal a larger number of hits in East Asia compared with Europe (East Asia summer precipitation = 72, Europe summer precipitation signals = 4; East Asia winter precipitation signals = 34, Europe winter precipitation signals = 3), while Europe reveals more hits for summer relative humidity than East Asia (East Asia summer relative humidity signals = 25, Europe summer relative humidity signals = 40) (Figure 3, Additional file 1: Table S3). By implication, the dominant forces driving adaptation differ between Europe and Asia.

## Discussion

We have implemented a novel approach for detecting signatures of recent natural selection in human populations in relation to climate, based on Mantel tests conducted in a general linear model framework. We compare our results with those of Hancock et al. (2011) [21], who use a Bayesian approach to find correlations between allele frequency and climate. We identify a number of candidate regions that exhibit significant agreement with Hancock et al. (2011)’s [21] findings, but our use of absolute rather than relative significance highlights order of magnitude differences in the number of hits found for the eight climate–season combinations. For example, summer and winter temperatures reveal associations with 0.013% and 0.27% of the genes, respectively. In terms of numbers of hits, winter solar radiation, winter temperature and summer precipitation seem to have exerted the greatest selective pressure on the human genome.

A perennial issue in any sort of genome-wide scan has been the necessity to control and correct for the large number of statistical tests performed, typically of the order of 10^6^. One way to reduce the problem is to analyze the genome as a series of usually non-overlapping windows and, within each, to maximize the signal by combining multiple, semi-independent measures of selection [38]. Our method offers an alternative way to reduce the multiple hypothesis testing problem. Through use of pairwise matrices, we are able to combine data from tens or even hundreds of SNPs within a genic window to yield a single relatedness value between any pair of individuals or populations. This reduces the number of tests conducted, largely dilutes the impact of occasional extreme outlier SNPs and, following Grossman et al., the use of relatedness effectively captures several consequences of selection that are usually tested separately. Use of similarity matrices further negates the need to fit specific models of inheritance, since regardless of how selection operates, virtually by definition, genetic distance among populations will be either greater (directional selection) or less (balancing selection) than expected based on the rest of the genome. Balancing selection in particular is often not tested for by classical approaches. However, balancing selection is expected to act on small genomic scales; restricting the 50 kb window size used here may improve the ability of MAGICS to accurately detect signals of balancing selection.

Approaches requiring extensive randomization are not usually implemented in genome-wide association (GWA) studies due to the prohibitive number of randomizations required to achieve a level of significance that indicates a locus of interest. Our approach offers two improvements. First, by combining data across multiple SNPs the total number of tests is reduced considerably, with a parallel decrease in the required minimum number of randomizations. Second, and more importantly, we introduce a data-splitting element, with the product of the P-values obtained in each half of the data providing an excellent predictor of overall significance. Through data-splitting, any given number of randomizations can estimate P-values that are approximately the square of the minimum P-value obtainable without splitting. This makes randomization a viable method for assessing significance even for genome-wide analyses.

The association of *ADRA2C* with cold temperatures (Figure 2) is supported by studies suggesting that the C subunit of the Alpha-2 Adrenergic Receptor is involved in vasoconstriction associated with response to cold [39]. In addition, polymorphisms in both *ADRA2A* and *ADRA2C* genes have been reported to play a role in modifications of cold and pain sensibility [40]. *ADRA2A* and *ADRA2C* seem to provide a clear example of climate driven selection, where independent regions of the genome have experienced selective pressure in human populations due to their role in cold and pain response. We speculate that, in contrast to the positive selection inferred at most other 'hits’, these loci have experienced balancing selection. Our argument is based on the rate at which heterozygosity declines with geographic distance from Africa in this region of the genome [30]. At both *ADRA2A* and *ADRA2C* the slope is appreciably shallower than expected, with *ADRA2C* being among the top 10% of regions with the most positive slopes, suggesting that selection has acted to retard the loss of diversity that occurred 'out of Africa’ (Additional file 1: Figure S3).

Previous studies [21] have tended to adopt a pragmatic approach to the interpretation of their results. Instead of trying to interpret the resulting P-values directly, allowing for the number of tests conducted, genetic non-independence between populations with shared ancestry as well as other confounding factors, P-values are ranked on the reasonable assumption that lower P-value on average indicate stronger candidates. Our method differs in that the use of non-parametric randomization allows reasonably objective 'absolute’ P-values to be generated. The approach of evaluating environmental variables separately has also been explored by Fumagalli et al., which also uses partial Mantel tests to examine correlations among genetic and climatic variation using a window-based approach (2011). That this offers a clear advantage is suggested by the contrasting results from the eight climate variables analyzed. Climate variable relative humidity, for example, yields very little evidence of selection, while others, most notably winter solar radiation, winter temperature, and summer precipitation, provide abundant evidence. This pattern seems biologically realistic, in that one would not expect selection to act equally on all variables. In contrast, ranking methods assign equal importance to all climate variables. The large difference between climate variables that we report also helps to validate our approach. One could argue that associations are inevitable given the high degree of autocorrelation between both genetic and climatic data. That some climate variables generate effectively no good hits is important because it suggests that our GLMs successfully control for autocorrelation such that the majority of hits found are genuine.

MAGICS differs in other ways from the method used to detect climate-specific selection signals in Hancock et al. (2011) [21]. In addition to differences in the unit of inference, specifically SNPs versus genic windows, we are also able to ask directly the source of significant associations. Fumagalli et al. (2011) [1] develop a similar strategy, but Hancock et al. (2011) [21] achieve this *post-hoc*. Our method has the flexibility to restrict randomization to subsets of the data. In practice, we start by randomizing across the entire dataset, then test for whole continent effects by restricting randomizations to within continents, and finally test for specific within-continent effects by randomizing within individual continents while holding all other data constant. This analysis sheds light on another facet of the patterns we detected, and may help to distinguish between associations that are due entirely to differences in climate between continents, and those that can arise due to selection in relation to other factors that become spuriously linked to the tested climate variables simply because two or more global regions either share or differ in their climate. Future iterations of our work may incorporate gene expression data, as it has been suggested to drive environmental adaptations in humans [41].

A concern of any association study is the possibility of false positives. Our results suggest that this issue has been largely mitigated. In isolation, a method prone to false positives due to aspects of the genetic data alone, for example linkage disequilibrium, ascertainment bias or genotyping errors, should generate similar numbers of 'hits’ with all climate variables. This is clearly not the case in our analyses, since relative humidity yields far fewer extreme values compared with all other variables and winter and summer seasons often yield very different numbers. The other concern is that the climate variables themselves tend to be distributed in a way that promotes spurious associations. For example, if people migrated along or against climatic clines, genetic similarity and climate could become correlated. That our winter and summer values usually generate very different numbers of hits despite being correlated with each other, would again seem to indicate that the 'hits’ are largely genuine.

## Conclusions

We demonstrate here that MAGICS is a powerful and flexible approach that can be used to identify regions of the genome involved in adaptations to specific environmental variables, isolating them from highly related confounding factors such as geographic distance, and also being able to localize the signals to particular regions of the world. The increasing availability of whole genome sequences of individuals from multiple global populations will provide additional opportunities to carefully study the specific influences of the environment on genomic variation.

## Methods

### Genetic data

We combined published data for 862 individuals belonging to 45 distinct global populations drawn from five published sources [42-46] (Additional file 1: Table S1). Only populations represented by a minimum of nine unrelated samples were included in the analysis, with the exception of the HGDP Colombian population, represented by 7 individuals, to increase the presence of populations from the Americas. We also merged North Italians and Tuscans from the data of Li et al. (2008), to provide a larger sample size for Italians. Several populations from India were merged to form a “South Indian” population.

Gene positions for the implementation of MAGICS were determined using NCBI Build 36.1, University of California Santa Cruz version Hg18. Ensembl gene prediction models recognize a total of 34,156 protein coding genes, RNA genes and pseudogenes in this build of the human genome. Among these, 28,784 were covered by at least one SNP and were used in our analyses. We constructed 'genic windows’ around these genes by taking the midpoint of each gene, calculated as (transcription start + end positions)/2, and the region 25 kb either side of this point. We chose to keep the windows of constant size to minimize bias in favor of larger genes, but acknowledge that cases can be made for a number of other strategies. All comparisons between our data set and that of Hancock et al. (2011) [21] were limited to the subset of 27,096 genes found in both studies.

### Geographic and climate data

Geographic coordinates of populations were taken from published data, where available, or else taken from the midpoint or capital of the country or region of origin. Land-only geographic separation in kilometers were provided by A. Manica [25]. To investigate the role of specific environmental variables on human genetic variation we focused on four climate variables and their variation across two seasons: (1) T, air temperature (measured in °C), (2) Pr, precipitation rate (kg/m^2^/s), (3) rH, relative humidity (%), and (4) Sr, solar radiation (W/m^2^). The data source we use reports data in terms of monthly averages across ~40 years of the collection, from the NCEP/NCAR database [47]. To account for seasonal variation, we took the summer and winter seasonal averages. Data extraction from the NCEP/NCAR website (http://ftp.cdc.noaa.gov/Datasets/ncep.reanalysis/) was conducted using an in house Perl script (Climate Manager), available upon request.

### Statistical analyses

MAGICS was implemented using a custom R script (http://cran.r-project.org). To maximize algorithm speed in order to conduct large numbers of randomizations, we exploited the R package Rcpp which facilitates easy incorporation of C++ code snippets. C++ subroutines were written to calculate linearized pairwise dissimilarity matrices from genetic/climate data. Pairwise F_ST_ values between all populations were pre-calculated using Weir and Cockerham’s 1984 estimator [48].

Significance was tested by extensive randomization. We chose to randomize the climate variable, in each case scrambling (sampling without replacement) the climate variable values among populations, recalculating the dissimilarity matrix and refitting the original model. Randomized fits that yielded lower Akaike Information Criterion (AIC) values than the original model were tallied. To maximize algorithm speed and to avoid spending large amounts of time on non-significant genomic regions, randomization number was increased initially up to a maximum of 100,000 until either 10 more extreme AIC values had been obtained from a minimum of 100 randomizations, whichever came first. Note that unless the randomization process ends without finding 10 more extreme AIC values, P-values tend to be slightly conservative due to the fact that they end on a success.

The simplest form of randomization involves scrambling the climate variable evenly across all populations. However, this strategy could increase the chance of weak associations appearing disproportionately strong if a climate value that appears to be found globally is actually unique to a single continent. For a more conservative strategy, we therefore repeated our analysis but this time restricted scrambling to within each of six pre-defined continents: Europe, East Asia, Central and South Asia, Middle East, Americas, and Oceania. Finally, we can also use our approach to ask which continents contribute most to any particular putative association. To do this, once a 'hit’ is found, the analysis is repeated for each continent in turn, scrambling data only for the current continent while holding all other data constant.

### Data-splitting methods

Randomization approaches benefit from being non-parametric but an importance disadvantage is that they are computer-intensive. This problem is particularly acute in GWA studies, where the determination of experiment-wide significance requires literally billions of iterations. We therefore explored methods of extrapolation. Trials based on the distribution of AIC values or the proportion of null deviance explained were unsuccessful due to long, poorly defined tails in the distribution of the randomized statistics. A more promising approach is based on data-splitting. If data for a significant regression are divided into two random halves, the P-value of the whole should approximate the product of the P-value of the two halves analyzed separately, an assertion that we verified by simulation. We therefore explored an algorithm in which, for each randomization, the dataset was divided into two random halves, each of which was then analyzed as a separate set of pairwise matrices. Specifically, two random halves are defined afresh at each randomization, used to analyze both the unscrambled and scrambled data, and counts tallied for each half when the AIC of the scrambled data yielded a lower AIC value than the unscrambled data. This approach was then applied to all highly significant 'hits’ (P = 0 in 10^5^ randomizations) but with the maximum randomization number extended to 2,000,000 and with full-dataset randomizations conducted in parallel for calibration.

### Data availability

The results from the MAGICS analyses for all the 28,784 genic windows are available online (http://www.zoo.cam.ac.uk/zoostaff/meg/MAGICSresults.xlsx). Information on the results table are provided in: The link http://www.zoo.cam.ac.uk/zoostaff/meg/README_Raj_et_al_2013.txt.

### Balancing versus positive selection

In MAGICS the coefficients of the GLM should be informative about the nature of the selection acting locally. If populations experiencing contrasting climates also exhibit greater than expected differentiation this would suggest directional selection. However, there is appreciable ambiguity. Where populations in similar climates are more genetically similar than expected this might either indicate directional selection fixing a similar variant or balancing selection reducing divergence through drift. For a clearer picture we therefore turned to a method based on variation in the extent to which variability was lost as humans migrated out of Africa.

Overall, heterozygosity declines with distance from Africa. However, the exact amount of variability lost should be modulated by natural selection, with balancing selection tending to reduce loss and directional selection tending to accelerate loss of heterozygosity. This concept has been used successfully to show that genomic regions that show signatures of selection are enriched for immune genes [30]. Inference of the mode of selection acting on a given genomic location was conducted exactly following Amos and Bryant (2011) [30], using their data. Briefly, Amos and Bryant (2011) reported the Pearson correlation coefficients of average SNP hetereozygosity and distant from Africa. Data comprise Phase II and III SNP data from HapMap, representing seven different populations and the analysis was conducted with a window size of 50 Kb. An argument could be made to calculate slopes using our data from 45 global populations. However, we elected not to do this for several reasons, including non-independent from other analyses and the fact that Phase I data may be subject to ascertainment bias. By using the data from Amos and Bryant (2011) [30] we achieve statistical independence from our data with a dataset that has proven ability to detect contrasting patterns of selection at immune-related genes. We therefore applied the same algorithm to derive the correlation coefficient and slope for the relationship between heterozygosity and distance from Africa for each of our genic windows, allowing us to define the mean and standard deviation, and hence to ask whether our putative 'hits’ show independent evidence of selection, and if so, whether the selection is likely to be balancing or directional.

### Supplemental data

The Supplemental Data section includes three figures and three tables.

### Web resources

Results for all the genic windows are available at: http://www.zoo.cam.ac.uk/zoostaff/meg/MAGICSresults.xlsx. Information on the results table are provided in: The link http://www.zoo.cam.ac.uk/zoostaff/meg/README_Raj_et_al_2013.txt.

## Competing interests

The authors would like to declare no competing financial or personal interests in the preparation of this manuscript.

## Authors’ contributions

WA, SR, and TK conceived and designed the experiments. WA, SR, LP and IGR analyzed the data, while WA developed and coded the MAGICS approach. SR, WA, TK, LP, IGR wrote the paper. All authors read and approved the final manuscript.

## Supplementary Material

Additional file 1: Figure S1 The number of genic windows showing P <= 10^-4^ (blue) or <= 10^-5^ (red) in more than 1,2 3 or 4 climate combinations 2. **Figure S2:** Regression line between the –log_10_P obtained using the full dataset run on 2*10^6^ randomizations (P_true_) and the same run masking half of the dataset each time (P_halves_) 2. **Figure S3:** Inference of directional and balancing selection associated with climate 3. **Table S1.** Climate variables assigned to the 45 populations used in this study 4. **Table S2:** Correlation of MAGIC hits by climate variables 5. **Table S3:** All the genic windows yielding a hit ( -log_10_P >=5) in the present study 7. Click here for file
