# Supplementary material for: A general linear model-based approach for inferring selection to climate
Source: BMC Genet. 2013 Sep 22;14:87. doi: 10.1186/1471-2156-14-87 (PMC3853933; doi:10.1186/1471-2156-14-87)
Supplement: Additional file 1: Figure S1 — The number of genic windows showing P <= 10-4 (blue) or <= 10-5 (red) in more than 1,2 3 or 4 climate combinations 2. Figure S2: Regression line between the –log10P obtained using the full dataset run on 2*106 randomizations (Ptrue) and the same run masking half of the dataset each time (Phalves) 2. Figure S3: Inference of directional and balancing selection associated with climate 3. Table S1. Climate variables assigned to the 45 populations used in this study 4. Table S2: Correlation of MAGIC hits by climate variables 5. Table S3: All the genic windows yielding a hit ( -log10P >=5) in the present study 7. [file 1471-2156-14-87-S1.docx]

Supplementary Data for the manuscript entitled,

**A Mantel test and general linear model based approach for inferring selection to climate**

Srilakshmi M. Raj, Luca Pagani, Irene Gallego Romero, Toomas Kivisild, William Amos

**Supplementary Figures and Tables**

**Figure S1.** The number of genic windows showing P <= 10^-4^ (blue) or

<= 10^-5^ (red) in more than 1,2 3 or 4 climate combinations…………………………………..2

**Figure S2.** Regression line between the –log_10_P obtained

using the full dataset run on 2*10^6^ randomizations (P_true_) and the same run

masking half of the dataset each time (P_halves_)………………………………………………...2

**Figure S3.** Inference of directional and balancing selection

associated with climate…………………………………………………………………………3

**Table S1.** Climate variables assigned to the 45 populations

used in this study………………………………………………………………………………..4

**Table S2.** Correlation of MAGIC hits by climate variables……………….5

**Table S3.** All the genic windows yielding a hit ( -log_10_P >=5)

in the present study……………………………………………………………………………..7

**Supplementary Figures**


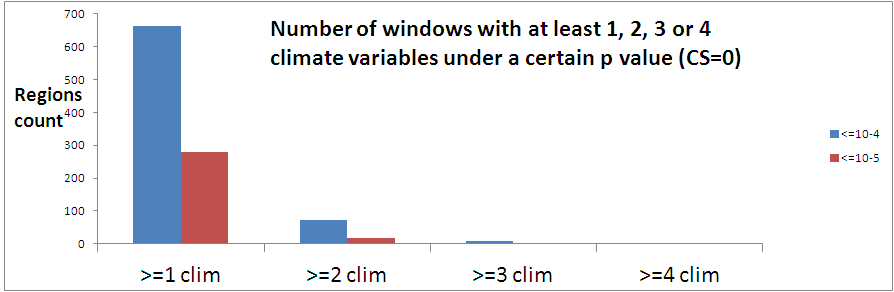


**Figure S1.** The number of genic windows showing P <= 10^-4^ (blue) or <= 10^-5^ (red) in more than 1,2 3 or 4 climate combinations.

**
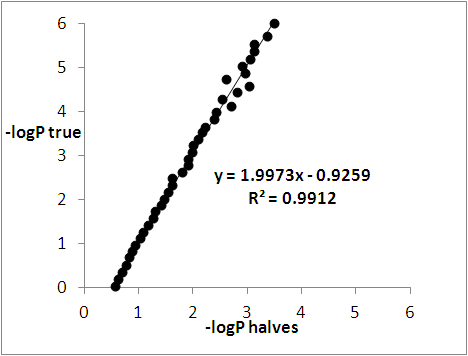
**

**Figure S2.** Regression line between the –log_10_P obtained using the full dataset run on 2*10^6^ randomizations (P_true_) and the same run masking half of the dataset each time (P_halves_). The displayed equation was used to infer the P_true_=0 after 2 * 10^6^ randomizations.


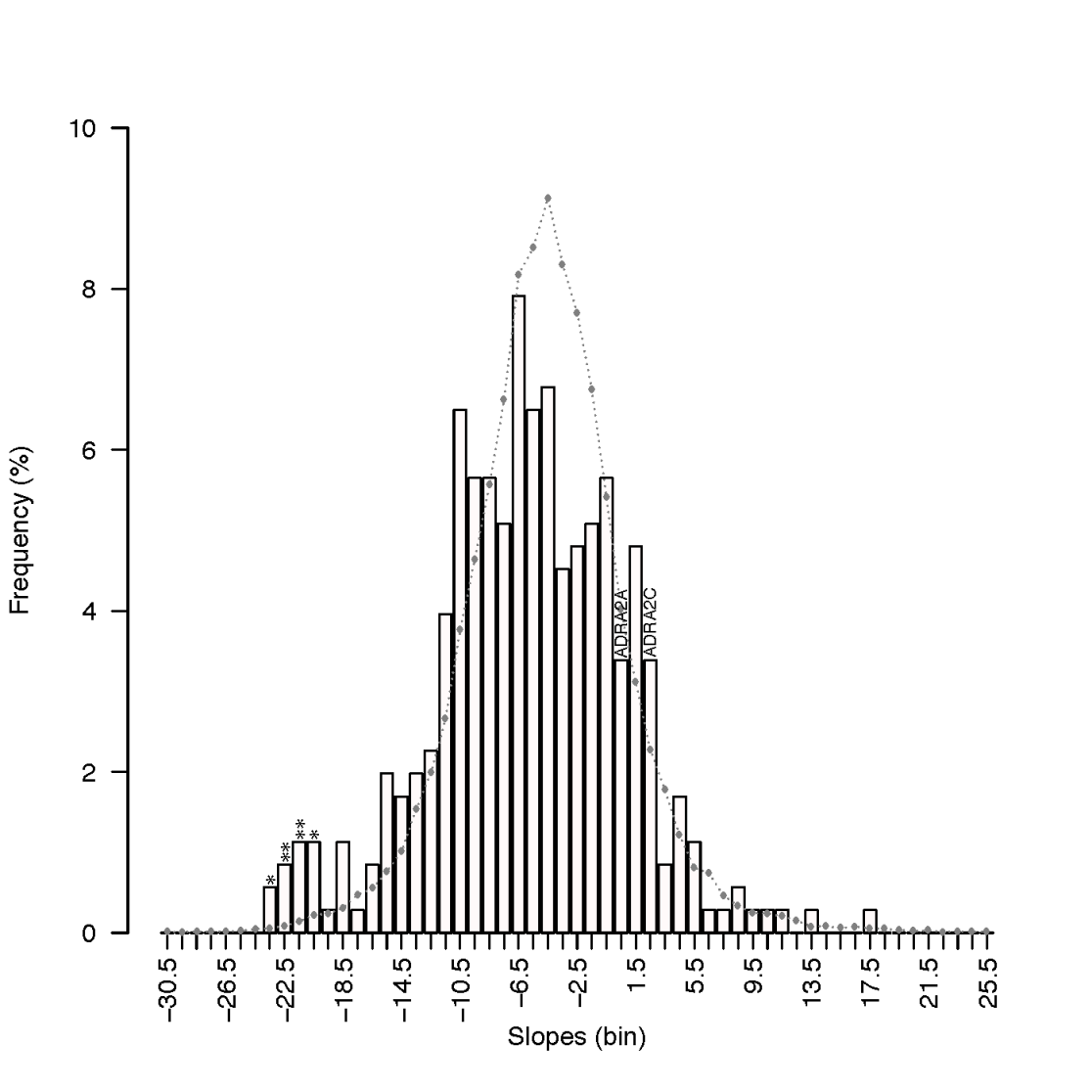


**Figure S3.** Inference of directional and balancing selection associated with climate. At any given locus, the slope of the relationship between heterozygosity and distance from Africa provides an indication of the nature of selection: neutral loci on average have negative slopes while those under balancing selection have zero or even slightly positive slopes while those under directional selection may have more extreme negative slopes. We plotted the frequency distribution of slopes for our hits (P<10-5, white bars) relative to the underlying distribution of slopes observed for all non-hit genes, indicated by the dotted line. Filled circles denote the frequency of non-hit genes at a given bin. Significance of excesses and deficits in each slope bin are obtained with a simple chi-squared test. Those bins that met significance are identified by asterisks: * denotes P>0.05, ** denotes P>0.01. While individual bin significance is achieved only among the most negative slopes, it is clear that our hits generally show an excess of 'neutral' slopes and a strong overall excess of strongly negative slope. Our two top hits, *ADRA2A* and *ADRA2C* both have slopes near zero, indicating balancing selection.

**Supplementary Tables**

**Table S1.** Climate variables assigned to the 45 populations used in this study.

|  |  |  |  |  |  | **Winter** | | | | **Summer** | | | |
| --- | --- | --- | --- | --- | --- | --- | --- | --- | --- | --- | --- | --- | --- |
| **Continent** | **Map Number** | **Population** | **N** | **long** | **lat** | **T** | **Sr** | **Pr** | **rH** | **T** | **Sr** | **Pr** | **rH** |
| 1. Europe and Altai |  |  |  |  |  |  |  |  |  |  |  |  |  |
|  | 1 | Orcadians | 15 | -9.65 | 60 | 5.7 | -35.1 | 10.5 | 80.9 | 12.4 | -118.5 | 19.8 | 86.4 |
|  | 2 | Spanish | 12 | -7.15 | 37.5 | 10.6 | -113.5 | 13.1 | 76.3 | 24.8 | -204.0 | 20.9 | 48.2 |
|  | 3 | French | 28 | 2.5 | 45 | 4.4 | -82.1 | 10.7 | 88.1 | 18.4 | -178.3 | 21.9 | 69.7 |
|  | 4 | Italians | 19 | 10 | 45 | 2.7 | -83.6 | 8.1 | 81.4 | 17.6 | -168.7 | 20.7 | 76.6 |
|  | 5 | Lithuanians | 10 | 22.5 | 55 | -1.4 | -35.9 | 8.5 | 89.7 | 16.2 | -116.3 | 22.6 | 78.9 |
|  | 6 | Romanians | 16 | 25 | 45 | -1.3 | -71.5 | 8.7 | 86.3 | 17.7 | -162.9 | 22.8 | 74.7 |
|  | 7 | Belorussians | 10 | 27.5 | 55 | -4.6 | -31.2 | 7.7 | 89.2 | 15.7 | -107.3 | 22.2 | 77.3 |
|  | 8 | Turks | 19 | 32.5 | 40 | -1.4 | -78.8 | 7.0 | 77.3 | 17.7 | -164.1 | 16.2 | 55.6 |
|  | 9 | Adygei | 17 | 40 | 45 | 2.3 | -74.2 | 10.3 | 87.1 | 21.5 | -170.7 | 25.4 | 66.5 |
|  | 10 | Mordovians | 15 | 45 | 55 | -7.9 | -28.1 | 6.7 | 89.2 | 16.2 | -113.2 | 22.6 | 76.0 |
|  | 11 | Armenians | 16 | 45 | 40 | -5.3 | -65.8 | 5.2 | 85.3 | 16.0 | -157.4 | 16.4 | 63.8 |
|  | 12 | Chuvashs | 17 | 47.5 | 55 | -8.7 | -29.9 | 6.5 | 89.1 | 16.6 | -117.6 | 22.5 | 73.0 |
|  | 13 | Turkmens | 15 | 57.5 | 37.5 | 3.1 | -88.4 | 7.8 | 55.4 | 25.7 | -177.4 | 13.7 | 17.7 |
|  | 14 | Altaians | 13 | 85 | 50 | -14.2 | -42.3 | 2.8 | 82.1 | 10.9 | -151.1 | 13.0 | 71.1 |
| 2. Middle East and North Africa |  |  |  |  |  |  |  |  |  |  |  |  |  |
|  | 15 | Mozabites | 27 | 2.5 | 32.5 | 11.7 | -99.9 | 9.9 | 44.5 | 30.8 | -151.6 | 17.9 | 17.7 |
|  | 16 | Egyptians | 12 | 30 | 30 | 14.6 | -128.0 | 13.0 | 61.6 | 26.3 | -196.0 | 19.4 | 60.6 |
|  | 17 | Palestinians | 46 | 35 | 32.5 | 13.1 | -94.5 | 12.8 | 71.1 | 24.5 | -155.3 | 20.9 | 76.4 |
|  | 18 | Syrians | 16 | 37.5 | 35 | 9.0 | -130.1 | 11.2 | 72.5 | 27.4 | -248.1 | 20.5 | 47.5 |
|  | 19 | Jordanians | 20 | 37.5 | 30 | 10.4 | -121.0 | 9.1 | 45.0 | 27.1 | -182.8 | 16.7 | 35.3 |
|  | 20 | Saudis | 20 | 45 | 25 | 16.5 | -124.8 | 14.6 | 37.1 | 34.2 | -189.3 | 16.6 | 12.7 |
|  | 21 | Iranians | 20 | 50 | 35 | -0.7 | -94.5 | 4.7 | 68.7 | 21.0 | -194.8 | 12.7 | 35.1 |
| 3. Central and South Asia |  |  |  |  |  |  |  |  |  |  |  |  |  |
|  | 22 | Balochi | 24 | 67.5 | 30 | 11.3 | -119.7 | 9.2 | 36.3 | 28.8 | -170.8 | 21.6 | 28.7 |
|  | 23 | Tajiks | 15 | 70 | 40 | -2.0 | -55.8 | 7.0 | 90.4 | 17.3 | -154.4 | 10.9 | 34.7 |
|  | 24 | Gujaratis | 88 | 72.5 | 22.5 | 23.8 | -148.5 | 13.6 | 28.7 | 26.2 | -130.9 | 47.4 | 82.9 |
|  | 25 | Burusho | 25 | 75 | 37.5 | -13.8 | -72.3 | 1.4 | 55.7 | 3.8 | -154.6 | 5.5 | 62.3 |
|  | 26 | South Indians | 19 | 77.5 | 12.5 | 24.5 | -183.9 | 23.5 | 55.3 | 22.7 | -155.0 | 40.8 | 84.2 |
| 4. East Asia |  |  |  |  |  |  |  |  |  |  |  |  |  |
|  | 27 | Uygurs | 10 | 80 | 45 | -4.9 | -50.6 | 6.0 | 84.6 | 17.1 | -149.3 | 15.0 | 45.7 |
|  | 28 | Tuvinians | 16 | 92.5 | 52.5 | -13.1 | -39.9 | 4.1 | 89.3 | 11.3 | -145.4 | 17.3 | 90.6 |
|  | 29 | Burmese | 15 | 95 | 22.5 | 19.3 | -139.6 | 13.8 | 34.2 | 23.0 | -114.7 | 45.9 | 93.5 |
|  | 30 | Tu | 10 | 100 | 35 | -11.6 | -108.6 | 2.6 | 72.4 | 7.3 | -188.1 | 11.8 | 78.4 |
|  | 31 | Nganassans | 15 | 102.5 | 75 | -27.1 | -4.2 | 2.5 | 76.6 | 3.4 | -73.6 | 13.7 | 90.3 |
|  | 32 | Cambodians | 10 | 105 | 12.5 | 26.9 | -155.0 | 38.2 | 61.1 | 25.1 | -135.4 | 51.2 | 93.9 |
|  | 33 | Evenkis | 16 | 107.5 | 55 | -17.1 | -27.9 | 2.9 | 82.6 | 12.5 | -120.8 | 18.4 | 79.6 |
|  | 34 | Mongolians | 9 | 110 | 45 | -12.3 | -65.5 | 3.2 | 78.2 | 19.9 | -147.5 | 17.1 | 38.0 |
|  | 35 | Han | 44 | 115 | 32.5 | 5.6 | -99.1 | 13.3 | 74.0 | 24.7 | -117.1 | 47.1 | 89.2 |
|  | 36 | Oroqens | 9 | 127.5 | 50 | -15.4 | -44.1 | 3.2 | 85.2 | 16.8 | -155.2 | 25.1 | 82.2 |
|  | 37 | Yakuts | 25 | 130 | 62.5 | -26.9 | -22.5 | 2.2 | 89.7 | 12.9 | -123.8 | 18.9 | 76.8 |
|  | 38 | Japanese | 28 | 137.5 | 37.5 | 1.2 | -111.1 | 7.1 | 80.7 | 21.5 | -160.2 | 35.8 | 87.4 |
|  | 39 | Koryaks | 18 | 160 | 57.5 | -10.3 | -24.9 | 4.3 | 90.5 | 10.3 | -123.9 | 18.9 | 88.4 |
| 5. Oceania |  |  |  |  |  |  |  |  |  |  |  |  |  |
|  | 40 | Papuans | 17 | 142.5 | -5 | 21.8 | -175.1 | 36.9 | 91.6 | 22.1 | -188.9 | 39.3 | 94.0 |
|  | 41 | Melanesians | 11 | 155 | -5 | 27.0 | -128.9 | 45.0 | 80.7 | 27.9 | -146.9 | 48.9 | 78.4 |
| 6. Native Americans |  |  |  |  |  |  |  |  |  |  |  |  |  |
|  | 42 | Pima | 14 | 252.5 | 30 | 10.7 | -119.2 | 7.2 | 36.4 | 23.4 | -143.1 | 25.3 | 53.9 |
|  | 43 | Mayas | 21 | 270 | 20 | 24.2 | -152.4 | 30.7 | 76.7 | 27.7 | -174.2 | 43.5 | 81.2 |
|  | 44 | Colombians | 7 | 292.5 | 2.5 | 23.6 | -145.1 | 44.7 | 95.4 | 23.7 | -151.1 | 41.9 | 92.5 |
|  | 45 | Karitiana | 13 | 297.5 | -10 | 25.5 | -122.1 | 36.6 | 71.6 | 24.1 | -119.2 | 46.9 | 95.6 |

The four climate variables we used are: (1) T, air temperature (measured in ºC), (2) Pr, precipitation rate (kg/m2/s), (3) rH, relative humidity (%), and (4) Sr, solar radiation (W/m2). The data source we use reports data in terms of monthly averages across ~40 years of the collection, from the NCEP/NCAR database: ftp://ftp.cdc.noaa.gov/Datasets/ncep.reanalysis/ ([Kistler et al. 2001](#_ENREF_1)).

Kistler, R., E. Kalnay, W. Collins, S. Saha, G. White *et al.*, 2001 The NCEP-NCAR 50-Year Reanalysis: Monthly Means CD-ROM and Documentation. Bulletin of the American Meteorological Society **82:** 247-268.

**Table S2.** Spearman rank correlation of MAGIC hits by climate variables.

|  | **Ts** | **Tw** | **Srs** | **Srw** | **Prs** | **Prw** | **rHs** | **rHw** |
| --- | --- | --- | --- | --- | --- | --- | --- | --- |
| **Ts** | 1 | 0.64 | 0.19 | 0.51 | 0.22 | 0.26 | 0.15 | 0.25 |
| **Tw** | 0.64 | 1 | 0.08 | 0.76 | 0.5 | 0.64 | 0 | 0.19 |
| **Srs** | 0.19 | 0.08 | 1 | 0.25 | 0.04 | 0 | 0.25 | 0.02 |
| **Srw** | 0.51 | 0.76 | 0.25 | 1 | 0.38 | 0.46 | 0 | 0.28 |
| **Prs** | 0.22 | 0.5 | 0.04 | 0.38 | 1 | 0.61 | 0.31 | 0.02 |
| **Prw** | 0.26 | 0.64 | 0 | 0.46 | 0.61 | 1 | 0.1 | 0 |
| **rHs** | 0.15 | 0 | 0.25 | 0 | 0.31 | 0.1 | 1 | 0.16 |
| **rHw** | 0.25 | 0.19 | 0.02 | 0.28 | 0.02 | 0 | 0.16 | 1 |
|  |  |  |  |  |  |  |  |  |

Tw - Winter temperature

Ts - Summer temperature

Prw - Winter precipitation

Prs - Summer precipitation

rHw - Winter relative humidity

rHs - Summer relative humidity

Srw - Winter solar radiation

Srs - Summer solar radiation

**Table S3.** All the genic windows yielding a hit ( -log_10_P >=5) in the present study.

| **-log_10_(P-split)**  **2*10^6^ rand.** | **Extrapolated**  **-log_10_(P)** | **ENSEMBL**  **GENE ID** | **HUGO GENE ID** | **Climate** | **Season** | **Continent Scrambling** | **Europe and Altai**  **p-value** | **Middle East and North Africa** | **Central and South Asia** | **East Asia** | **Oceania** | **Americas** | Slope of Heterozygosity /Geographic Distance from Addis Ababa | Correlation Coefficient of Heterozygosity/  Geographic Distance from Addis Ababa |
| --- | --- | --- | --- | --- | --- | --- | --- | --- | --- | --- | --- | --- | --- | --- |
| 5.52 | 10.10 | ENSG00000150594 | ADRA2A | Sr | Winter | 0 | 2.50E-01 | 2.10E-01 | 1.50E-02 | 7.90E-03 | 0.50 | 0.81 | 0.17 | 0.01 |
| 4.92 | 8.90 | ENSG00000184160 | ADRA2C | T | Winter | 0 | 4.67E-02 | 2.10E-01 | 4.94E-03 | 5.93E-03 | 0.51 | 0.16 | 2.65 | 0.20 |
| 4.77 | 8.60 | ENSG00000207207 | #NA# | Sr | Winter | 0 | 3.80E-01 | 2.80E-01 | 2.91E-02 | 2.80E-05 | 0.53 | 0.83 | -9.42 | -0.54 |
| 4.77 | 8.60 | ENSG00000213247 | #NA# | Sr | Winter | 0 | 1.00E-01 | 2.20E-01 | 7.55E-03 | 6.46E-04 | 0.45 | 0.96 | 2.03 | 0.16 |
| 4.62 | 8.30 | ENSG00000213247 | #NA# | Pr | Winter | 0 | 4.46E-02 | 1.00E-01 | 4.90E-02 | 6.61E-03 | 1.00 | 0.63 | 2.03 | 0.16 |
| 4.55 | 8.17 | ENSG00000070886 | EPHA8 | Sr | Winter | 0 | 4.42E-02 | 6.90E-01 | 1.20E-01 | 1.61E-03 | 1.00 | 0.26 | -0.82 | -0.05 |
| 4.54 | 8.14 | ENSG00000209656 | #NA# | Sr | Winter | 0 | 4.87E-03 | 1.40E-01 | 5.10E-01 | 3.59E-04 | 1.00 | 0.81 | 2.06 | 0.09 |
| 4.52 | 8.11 | ENSG00000186329 | TMEM212 | rH | Summer | 0 | 1.02E-02 | 6.20E-01 | 6.00E-02 | 7.61E-03 | 0.42 | 0.19 | -10.61 | -0.73 |
| 4.49 | 8.05 | ENSG00000216328 | #NA# | rH | Winter | 0 | 9.00E-02 | 4.20E-01 | 7.00E-02 | 4.24E-02 | 0.54 | 0.54 | 1.55 | 0.09 |
| 4.46 | 7.97 | ENSG00000077514 | POLD3 | Sr | Winter | 0 | 2.20E-01 | 5.30E-01 | 8.36E-03 | 6.03E-04 | 0.44 | 0.61 | -2.39 | -0.18 |
| 4.46 | 7.97 | ENSG00000205243 | #NA# | Sr | Winter | 0 | 2.52E-03 | 1.32E-02 | 1.10E-01 | 4.90E-04 | 1.00 | 0.07 | -1.05 | -0.09 |
| 4.44 | 7.95 | ENSG00000060138 | CSDA | Pr | Summer | 0 | 9.90E-01 | 5.80E-01 | 8.00E-02 | 7.78E-04 | 1.00 | 0.04 | 7.87 | 0.51 |
| 4.39 | 7.84 | ENSG00000115738 | ID2 | rH | Summer | 0 | 7.31E-03 | 1.40E-01 | 8.00E-02 | 1.50E-01 | 0.50 | 0.22 | -10.10 | -0.58 |
| 4.36 | 7.78 | ENSG00000209656 | #NA# | T | Summer | 0 | 1.00E-01 | 1.50E-01 | 6.00E-01 | 4.95E-02 | 0.49 | 0.83 | 2.06 | 0.09 |
| 4.34 | 7.74 | ENSG00000163281 | GNPDA2 | Pr | Summer | 0 | 4.20E-01 | 6.10E-01 | 6.80E-01 | 1.14E-03 | 0.48 | 0.58 | 17.56 | 0.71 |
| 4.31 | 7.68 | ENSG00000198669 | #NA# | Pr | Summer | 0 | 4.40E-01 | 4.90E-01 | 2.10E-01 | 9.01E-03 | 1.00 | 0.32 | -18.85 | -0.69 |
| 4.23 | 7.52 | ENSG00000144476 | CXCR7 | rH | Summer | 0 | 1.00E-01 | 9.00E-02 | 6.00E-02 | 1.20E-01 | 1.00 | 0.56 | -12.53 | -0.60 |
| 4.22 | 7.51 | ENSG00000139263 | LRIG3 | Pr | Winter | 0 | 1.00E+00 | 8.00E-01 | 2.20E-01 | 1.81E-02 | 0.47 | 0.04 | 2.77 | 0.23 |
| 4.22 | 7.51 | ENSG00000129347 | KRI1 | Pr | Summer | 0 | 8.53E-04 | 6.80E-01 | 1.50E-01 | 4.78E-04 | 0.50 | 0.19 | -8.73 | -0.53 |
| 4.21 | 7.48 | ENSG00000092470 | WDR76 | rH | Summer | 0 | 6.57E-04 | 3.10E-01 | 9.00E-02 | 3.22E-03 | 1.00 | 0.81 | -21.14 | -0.72 |
| 4.20 | 7.46 | ENSG00000075891 | PAX2 | rH | Summer | 0 | 2.70E-01 | 1.70E-01 | 8.00E-02 | 3.88E-04 | 1.00 | 0.92 | -20.24 | -0.65 |
| 4.20 | 7.46 | ENSG00000171877 | FRMD5 | rH | Summer | 0 | 1.63E-03 | 4.20E-01 | 4.80E-01 | 6.00E-02 | 1.00 | 0.27 | -14.41 | -0.63 |
| 4.19 | 7.45 | ENSG00000152292 | SH2D6 | Sr | Winter | 0 | 2.10E-01 | 2.50E-01 | 5.00E-02 | 2.26E-04 | 0.47 | 0.81 | -9.60 | -0.57 |
| 4.19 | 7.45 | ENSG00000221947 | XKR9 | Pr | Summer | 0 | 6.00E-01 | 5.40E-01 | 2.20E-01 | 4.84E-03 | 1.00 | 0.34 | -19.18 | -0.69 |
| 4.17 | 7.41 | ENSG00000199452 | #NA# | rH | Summer | 0 | 4.24E-02 | 5.00E-02 | 2.00E-01 | 1.20E-01 | 0.59 | 0.09 | -13.39 | -0.57 |
| 4.14 | 7.35 | ENSG00000206974 | #NA# | rH | Winter | 0 | 6.00E-02 | 3.70E-01 | 7.00E-02 | 7.00E-02 | 0.55 | 0.49 | 2.82 | 0.17 |
| 4.14 | 7.34 | ENSG00000208922 | #NA# | rH | Winter | 0 | 1.20E-01 | 5.20E-01 | 7.00E-02 | 8.00E-02 | 0.44 | 0.45 | -0.10 | -0.01 |
| 4.09 | 7.24 | ENSG00000214192 | UBE2V1P2 | rH | Summer | 0 | 6.94E-03 | 9.00E-02 | 4.50E-01 | 1.94E-02 | 1.00 | 0.25 | -12.28 | -0.55 |
| 4.03 | 7.12 | ENSG00000112335 | SNX3 | rH | Winter | 0 | 7.00E-02 | 4.30E-01 | 5.00E-02 | 1.10E-01 | 0.47 | 0.46 | 1.29 | 0.08 |
| 4.01 | 7.09 | ENSG00000201416 | SNORA71B | rH | Summer | 0 | 1.67E-02 | 2.84E-02 | 2.90E-01 | 5.00E-02 | 0.40 | 0.09 | -12.76 | -0.62 |
| 3.97 | 7.01 | ENSG00000031081 | ARHGAP31 | Sr | Winter | 0 | 3.50E-01 | 4.40E-01 | 3.33E-02 | 3.17E-03 | 0.46 | 0.20 | -0.34 | -0.03 |
| 3.93 | 6.92 | ENSG00000198284 | NUP210P1 | Pr | Summer | 0 | 1.80E-01 | 1.37E-02 | 5.00E-02 | 4.30E-03 | 1.00 | 0.08 | 1.24 | 0.10 |
| 3.92 | 6.90 | ENSG00000144802 | NFKBIZ | Sr | Winter | 0 | 1.18E-02 | 7.00E-02 | 2.40E-01 | 1.58E-02 | 1.00 | 0.34 | -6.88 | -0.49 |
| 3.91 | 6.89 | ENSG00000168781 | PPIP5K1 | rH | Summer | 0 | 3.17E-03 | 1.60E-01 | 5.70E-03 | 5.00E-02 | 1.00 | 0.50 | -15.41 | -0.59 |
| 3.90 | 6.87 | ENSG00000196564 | #NA# | rH | Summer | 0 | 5.98E-04 | 3.40E-01 | 8.00E-02 | 9.65E-03 | 1.00 | 0.31 | -16.73 | -0.69 |
| 3.89 | 6.85 | ENSG00000146233 | CYP39A1 | Sr | Winter | 0 | 4.72E-03 | 7.90E-01 | 2.20E-01 | 1.90E-01 | 0.51 | 0.57 | -3.84 | -0.31 |
| 3.87 | 6.80 | ENSG00000133226 | SRRM1 | T | Winter | 0 | 3.63E-03 | 1.51E-02 | 7.30E-01 | 3.41E-03 | 0.49 | 0.90 | 4.57 | 0.15 |
| 3.87 | 6.80 | ENSG00000144802 | NFKBIZ | T | Winter | 0 | 1.28E-02 | 4.80E-01 | 4.90E-01 | 6.00E-03 | 0.42 | 0.78 | -6.88 | -0.49 |
| 3.87 | 6.80 | ENSG00000201358 | #NA# | Pr | Summer | 0 | 1.20E-01 | 6.00E-01 | 2.29E-02 | 5.44E-03 | 0.45 | 0.02 | -10.91 | -0.73 |
| 3.86 | 6.78 | ENSG00000160221 | C21orf33 | Pr | Winter | 0 | 1.00E-01 | 3.70E-01 | 3.85E-02 | 1.76E-03 | 0.42 | 0.09 | 0.67 | 0.04 |
| 3.85 | 6.77 | ENSG00000208500 | #NA# | rH | Summer | 0 | 7.08E-03 | 1.00E-01 | 3.30E-01 | 2.87E-02 | 1.00 | 0.14 | -16.21 | -0.61 |
| 3.83 | 6.72 | ENSG00000222357 | #NA# | T | Winter | 0 | 1.40E-02 | 6.00E-02 | 1.30E-01 | 9.00E-02 | 0.44 | 0.38 | 0.08 | 0.01 |
| 3.80 | 6.67 | ENSG00000205497 | OR51A4 | Sr | Winter | 0 | 1.90E-01 | 1.80E-01 | 3.10E-01 | 3.16E-03 | 0.42 | 0.25 | -6.59 | -0.66 |
| 3.78 | 6.62 | ENSG00000208642 | #NA# | Pr | Winter | 0 | 1.30E-01 | 3.60E-01 | 9.00E-02 | 9.73E-03 | 1.00 | 0.39 | -1.90 | -0.14 |
| 3.77 | 6.61 | ENSG00000169618 | PROKR1 | rH | Summer | 0 | 1.50E-01 | 5.10E-01 | 5.00E-02 | 4.90E-02 | 1.00 | 0.04 | -10.78 | -0.52 |
| 3.74 | 6.55 | ENSG00000129460 | NGDN | Pr | Winter | 0 | 9.80E-01 | 6.00E-02 | 1.60E-01 | 4.90E-02 | 0.48 | 0.03 | 13.76 | 0.62 |
| 3.74 | 6.54 | ENSG00000214802 | #NA# | Pr | Summer | 0 | 1.30E-01 | 2.60E-01 | 5.00E-02 | 2.87E-03 | 0.53 | 0.04 | -8.81 | -0.32 |
| 3.74 | 6.54 | ENSG00000201599 | SNORA71A | rH | Summer | 0 | 5.00E-02 | 8.00E-02 | 2.10E-01 | 1.00E-01 | 0.53 | 0.03 | -11.96 | -0.56 |
| 3.73 | 6.52 | ENSG00000182035 | ADIG | rH | Summer | 0 | 1.80E-03 | 3.60E-01 | 3.00E-01 | 2.10E-01 | 0.44 | 0.44 | -8.59 | -0.41 |
| 3.72 | 6.50 | ENSG00000198128 | OR2L3 | T | Winter | 0 | 8.09E-03 | 1.00E+00 | 4.20E-01 | 2.14E-04 | 0.49 | 0.21 | -3.60 | -0.18 |
| 3.72 | 6.50 | ENSG00000113249 | HAVCR1 | Pr | Summer | 0 | 3.50E-01 | 1.10E-01 | 4.40E-01 | 2.29E-04 | 0.54 | 0.63 | -9.04 | -0.49 |
| 3.71 | 6.48 | ENSG00000209915 | #NA# | Sr | Winter | 0 | 2.86E-02 | 3.00E-01 | 7.00E-02 | 3.41E-03 | 0.37 | 0.21 | -5.64 | -0.35 |
| 3.70 | 6.47 | ENSG00000207188 | #NA# | rH | Summer | 0 | 2.32E-03 | 9.30E-01 | 3.03E-02 | 2.60E-01 | 1.00 | 0.20 | -9.95 | -0.49 |
| 3.70 | 6.46 | ENSG00000178950 | GAK | Pr | Winter | 0 | 6.20E-01 | 3.30E-01 | 6.60E-01 | 6.67E-04 | 1.00 | 0.21 | -8.15 | -0.66 |
| 3.69 | 6.45 | ENSG00000129636 | ITFG1 | Pr | Winter | 0 | 3.21E-02 | 1.00E-01 | 2.20E-01 | 2.28E-02 | 1.00 | 0.06 | -5.40 | -0.32 |
| 3.69 | 6.44 | ENSG00000150594 | ADRA2A | Pr | Summer | 0 | 9.40E-01 | 3.80E-01 | 6.00E-02 | 1.84E-03 | 1.00 | 0.37 | 0.17 | 0.01 |
| 3.68 | 6.43 | ENSG00000176105 | YES1 | Sr | Winter | 0 | 1.80E-01 | 1.90E-01 | 2.70E-01 | 9.30E-05 | 1.00 | 0.06 | 2.84 | 0.15 |
| 3.68 | 6.43 | ENSG00000211064 | #NA# | rH | Summer | 0 | 2.91E-02 | 2.76E-02 | 2.20E-01 | 9.00E-02 | 0.48 | 0.38 | -10.80 | -0.62 |
| 3.68 | 6.42 | ENSG00000163121 | NEURL3 | Sr | Winter | 0 | 6.00E-02 | 5.10E-01 | 4.40E-01 | 4.13E-02 | 0.56 | 0.46 | -12.56 | -0.61 |
| 3.67 | 6.41 | ENSG00000219683 | #NA# | Pr | Summer | 0 | 8.40E-01 | 8.30E-01 | 1.20E-01 | 1.33E-03 | 0.56 | 0.05 | 2.08 | 0.19 |
| 3.66 | 6.39 | ENSG00000219131 | #NA# | Pr | Summer | 0 | 4.00E-01 | 6.00E-01 | 2.10E-01 | 1.28E-04 | 0.52 | 0.24 | 6.74 | 0.44 |
| 3.66 | 6.38 | ENSG00000117133 | RPF1 | T | Winter | 0 | 2.20E-01 | 9.40E-01 | 5.32E-03 | 7.91E-04 | 1.00 | 0.21 | 4.26 | 0.19 |
| 3.65 | 6.37 | ENSG00000210389 | #NA# | Sr | Winter | 0 | 1.00E-01 | 9.30E-01 | 2.10E-02 | 5.21E-04 | 1.00 | 0.85 | 1.99 | 0.15 |
| 3.64 | 6.35 | ENSG00000207077 | #NA# | Pr | Summer | 0 | 2.70E-01 | 4.40E-01 | 6.00E-02 | 5.42E-03 | 0.51 | 0.11 | -8.46 | -0.40 |
| 3.64 | 6.34 | ENSG00000176105 | YES1 | T | Winter | 0 | 2.30E-01 | 5.20E-01 | 2.40E-01 | 7.35E-03 | 0.58 | 0.26 | 2.84 | 0.15 |
| 3.63 | 6.33 | ENSG00000189127 | ANKRD34B | rH | Summer | 0 | 1.66E-02 | 1.90E-01 | 5.00E-02 | 1.10E-01 | 1.00 | 0.44 | -14.64 | -0.73 |
| 3.63 | 6.31 | ENSG00000070886 | EPHA8 | T | Winter | 0 | 6.88E-03 | 4.10E-01 | 2.90E-01 | 7.47E-04 | 0.49 | 0.70 | -0.82 | -0.05 |
| 3.60 | 6.27 | ENSG00000214688 | C10orf105 | Sr | Winter | 0 | 4.81E-02 | 3.10E-01 | 6.70E-01 | 2.00E-06 | 1.00 | 0.68 | -1.43 | -0.19 |
| 3.60 | 6.27 | ENSG00000204611 | ZNF616 | Sr | Winter | 0 | 6.00E-02 | 9.50E-01 | 6.33E-03 | 7.46E-04 | 1.00 | 0.79 | 1.57 | 0.11 |
| 3.59 | 6.25 | ENSG00000216450 | #NA# | rH | Summer | 0 | 2.30E-01 | 2.69E-02 | 3.27E-02 | 1.00E-01 | 1.00 | 0.92 | -22.94 | -0.70 |
| 3.59 | 6.24 | ENSG00000211394 | #NA# | Pr | Summer | 0 | 2.10E-01 | 4.40E-01 | 2.84E-02 | 8.15E-04 | 0.46 | 0.05 | -9.37 | -0.34 |
| 3.58 | 6.22 | ENSG00000207310 | #NA# | Sr | Winter | 0 | 3.60E-01 | 4.60E-01 | 2.79E-02 | 1.08E-03 | 0.51 | 0.26 | -2.09 | -0.20 |
| 3.58 | 6.22 | ENSG00000137710 | RDX | Sr | Winter | 1 | 1.60E-01 | 1.30E-01 | 2.25E-02 | 1.05E-04 | 1.00 | 0.14 | -23.33 | -0.74 |
| 3.56 | 6.18 | ENSG00000102539 | MLNR | Pr | Winter | 0 | 1.30E-01 | 3.57E-02 | 6.30E-01 | 1.34E-02 | 0.50 | 0.05 | -8.52 | -0.43 |
| 3.55 | 6.17 | ENSG00000211371 | #NA# | Pr | Summer | 0 | 1.70E-01 | 3.20E-01 | 1.30E-01 | 3.29E-03 | 0.45 | 0.04 | -7.55 | -0.34 |
| 3.53 | 6.12 | ENSG00000107186 | MPDZ | Pr | Summer | 0 | 7.60E-01 | 7.30E-01 | 6.37E-03 | 1.22E-03 | 1.00 | 0.18 | -1.71 | -0.13 |
| 3.53 | 6.12 | ENSG00000085117 | CD82 | rH | Summer | 0 | 7.00E-02 | 4.90E-01 | 8.00E-02 | 3.55E-03 | 0.48 | 0.10 | -10.14 | -0.69 |
| 3.53 | 6.12 | ENSG00000140391 | TSPAN3 | Pr | Summer | 0 | 6.50E-01 | 1.30E-01 | 1.90E-01 | 1.36E-02 | 0.45 | 0.04 | -4.49 | -0.16 |
| 3.52 | 6.10 | ENSG00000115507 | OTX1 | rH | Summer | 0 | 1.20E-01 | 8.60E-01 | 4.59E-02 | 3.82E-02 | 0.52 | 0.29 | -2.59 | -0.19 |
| 3.51 | 6.08 | ENSG00000101189 | C20orf20 | Pr | Summer | 0 | 1.00E+00 | 6.80E-01 | 1.80E-01 | 1.50E-05 | 0.56 | 0.20 | -6.53 | -0.51 |
| 3.50 | 6.07 | ENSG00000221296 | MIR548T | Sr | Winter | 0 | 1.29E-02 | 3.33E-02 | 6.00E-02 | 1.41E-02 | 0.49 | 0.68 | 3.08 | 0.16 |
| 3.50 | 6.07 | ENSG00000206687 | #NA# | T | Winter | 0 | 4.76E-02 | 5.70E-01 | 1.30E-01 | 1.58E-02 | 0.54 | 0.32 | 5.41 | 0.39 |
| 3.49 | 6.05 | ENSG00000205496 | OR51A2 | Sr | Winter | 0 | 1.10E-01 | 1.50E-01 | 2.60E-01 | 5.10E-03 | 0.45 | 0.27 | -5.21 | -0.49 |
| 3.48 | 6.02 | ENSG00000186847 | KRT14 | rH | Summer | 0 | 1.92E-02 | 1.80E-01 | 5.00E-02 | 2.17E-02 | 0.47 | 0.64 | -21.46 | -0.70 |
| 3.47 | 6.00 | ENSG00000182168 | UNC5C | Pr | Summer | 0 | 7.30E-01 | 6.10E-01 | 3.10E-01 | 2.18E-03 | 0.58 | 0.09 | -10.19 | -0.53 |
| 3.46 | 5.99 | ENSG00000221502 | MIR1245 | rH | Summer | 0 | 1.44E-02 | 2.42E-02 | 4.50E-02 | 8.40E-01 | 1.00 | 0.84 | -4.26 | -0.37 |
| 3.46 | 5.98 | ENSG00000170035 | UBE2E3 | Sr | Winter | 0 | 9.00E-02 | 4.60E-01 | 1.30E-01 | 9.31E-03 | 1.00 | 0.91 | -15.54 | -0.62 |
| 3.46 | 5.98 | ENSG00000172893 | DHCR7 | Sr | Winter | 0 | 3.70E-01 | 4.10E-01 | 1.28E-02 | 1.27E-03 | 0.55 | 0.07 | 3.31 | 0.19 |
| 3.45 | 5.97 | ENSG00000103018 | CYB5B | rH | Summer | 0 | 3.10E-03 | 2.40E-01 | 2.16E-02 | 1.70E-01 | 1.00 | 1.00 | -3.17 | -0.35 |
| 3.45 | 5.96 | ENSG00000005810 | MYCBP2 | Sr | Winter | 0 | 3.31E-02 | 5.40E-01 | 6.00E-02 | 2.20E-04 | 0.39 | 0.22 | -6.79 | -0.28 |
| 3.44 | 5.95 | ENSG00000160220 | #NA# | Sr | Winter | 0 | 3.10E-01 | 6.50E-01 | 5.00E-02 | 1.19E-02 | 1.00 | 0.31 | 5.29 | 0.22 |
| 3.44 | 5.94 | ENSG00000209449 | #NA# | Pr | Winter | 0 | 7.00E-01 | 8.50E-01 | 2.50E-01 | 1.40E-01 | 0.46 | 0.03 | -0.38 | -0.04 |
| 3.44 | 5.94 | ENSG00000167664 | TMIGD2 | Pr | Winter | 0 | 3.50E-01 | 8.80E-01 | 1.80E-01 | 1.30E-01 | 0.49 | 0.06 | -4.73 | -0.49 |
| 3.43 | 5.92 | ENSG00000209362 | #NA# | Sr | Winter | 0 | 2.10E-01 | 4.70E-01 | 3.09E-02 | 6.26E-04 | 0.52 | 0.76 | -2.95 | -0.22 |
| 3.42 | 5.91 | ENSG00000201811 | #NA# | rH | Summer | 0 | 5.00E-02 | 1.00E-01 | 2.20E-01 | 8.00E-02 | 0.56 | 0.04 | -11.28 | -0.54 |
| 3.42 | 5.90 | ENSG00000137693 | YAP1 | T | Winter | 0 | 1.40E-01 | 9.00E-01 | 3.90E-01 | 6.89E-04 | 0.48 | 0.05 | -2.85 | -0.16 |
| 3.42 | 5.90 | ENSG00000210787 | #NA# | Sr | Winter | 0 | 1.82E-03 | 5.80E-01 | 2.40E-01 | 1.50E-03 | 0.57 | 0.60 | -3.96 | -0.32 |
| 3.42 | 5.90 | ENSG00000109851 | DBX1 | Sr | Winter | 0 | 4.59E-02 | 3.60E-01 | 1.17E-02 | 7.22E-03 | 0.40 | 0.98 | -0.69 | -0.08 |
| 3.41 | 5.89 | ENSG00000158486 | DNAH3 | T | Summer | 0 | 1.45E-02 | 1.90E-01 | 2.44E-02 | 1.39E-02 | 0.55 | 0.16 | 3.65 | 0.24 |
| 3.41 | 5.88 | ENSG00000212145 | #NA# | rH | Summer | 0 | 1.65E-03 | 7.00E-02 | 1.00E-01 | 3.03E-02 | 1.00 | 0.43 | -15.17 | -0.52 |
| 3.41 | 5.88 | ENSG00000185162 | #NA# | T | Winter | 0 | 6.08E-03 | 4.10E-01 | 3.00E-01 | 1.22E-02 | 0.43 | 0.60 | -2.13 | -0.25 |
| 3.40 | 5.87 | ENSG00000114302 | PRKAR2A | Sr | Winter | 0 | 1.20E-01 | 8.40E-01 | 2.14E-02 | 1.85E-04 | 0.53 | 0.25 | -6.94 | -0.30 |
| 3.38 | 5.83 | ENSG00000176105 | YES1 | Pr | Winter | 0 | 4.50E-01 | 7.00E-01 | 2.10E-01 | 3.43E-03 | 0.59 | 0.18 | 2.84 | 0.15 |
| 3.38 | 5.82 | ENSG00000216131 | #NA# | Pr | Winter | 0 | 3.30E-01 | 2.20E-01 | 6.00E-02 | 1.00E-01 | 1.00 | 0.29 | -11.35 | -0.68 |
| 3.38 | 5.82 | ENSG00000113761 | ZNF346 | Pr | Winter | 0 | 7.00E-02 | 6.70E-01 | 3.42E-02 | 2.12E-02 | 1.00 | 0.11 | -9.75 | -0.71 |
| 3.37 | 5.81 | ENSG00000100558 | PLEK2 | Pr | Summer | 0 | 9.20E-01 | 8.40E-01 | 3.76E-02 | 3.11E-03 | 1.00 | 0.72 | 10.06 | 0.48 |
| 3.36 | 5.78 | ENSG00000198832 | #NA# | Pr | Winter | 0 | 7.90E-01 | 5.80E-01 | 9.00E-02 | 2.00E-03 | 1.00 | 0.12 | -7.43 | -0.61 |
| 3.36 | 5.78 | ENSG00000217334 | #NA# | Sr | Winter | 0 | 5.72E-03 | 9.80E-01 | 1.90E-01 | 1.10E-01 | 0.52 | 0.24 | -18.28 | -0.51 |
| 3.35 | 5.77 | ENSG00000220066 | #NA# | Pr | Winter | 0 | 7.90E-01 | 5.50E-01 | 8.00E-02 | 8.87E-03 | 1.00 | 0.07 | -6.93 | -0.60 |
| 3.34 | 5.74 | ENSG00000198492 | YTHDF2 | rH | Summer | 0 | 4.50E-01 | 4.70E-01 | 1.00E-01 | 2.56E-03 | 0.50 | 0.76 | -11.56 | -0.55 |
| 3.33 | 5.73 | ENSG00000100362 | PVALB | Sr | Winter | 0 | 2.45E-03 | 5.30E-01 | 1.50E-01 | 3.23E-04 | 0.40 | 0.39 | -0.02 | 0.00 |
| 3.33 | 5.72 | ENSG00000216892 | #NA# | T | Winter | 0 | 1.80E-01 | 8.40E-01 | 2.70E-01 | 8.59E-03 | 0.51 | 0.13 | -3.26 | -0.28 |
| 3.32 | 5.71 | ENSG00000119203 | CPSF3 | rH | Summer | 0 | 3.50E-01 | 3.30E-01 | 2.92E-02 | 1.86E-03 | 0.51 | 0.65 | -21.49 | -0.68 |
| 3.32 | 5.71 | ENSG00000218638 | #NA# | rH | Summer | 0 | 2.60E-01 | 7.00E-02 | 2.35E-02 | 1.30E-01 | 1.00 | 0.94 | -23.30 | -0.71 |
| 3.32 | 5.70 | ENSG00000202487 | #NA# | Sr | Winter | 0 | 1.78E-02 | 1.30E-01 | 8.00E-02 | 2.30E-05 | 1.00 | 0.92 | -9.15 | -0.69 |
| 3.32 | 5.70 | ENSG00000206761 | #NA# | T | Winter | 0 | 7.40E-03 | 2.11E-02 | 2.72E-02 | 3.12E-03 | 0.46 | 0.28 | -1.78 | -0.14 |
| 3.31 | 5.68 | ENSG00000211732 | TRBV6-9 | rH | Summer | 0 | 7.28E-03 | 1.86E-02 | 3.60E-01 | 6.38E-03 | 0.55 | 0.91 | -5.58 | -0.48 |
| 3.31 | 5.68 | ENSG00000111206 | FOXM1 | Sr | Winter | 0 | 5.00E-02 | 4.50E-01 | 6.00E-02 | 4.24E-04 | 0.48 | 0.95 | -2.15 | -0.13 |
| 3.30 | 5.67 | ENSG00000203875 | SNHG5 | Sr | Winter | 0 | 2.36E-03 | 9.30E-01 | 2.50E-02 | 5.00E-02 | 0.56 | 0.52 | -22.03 | -0.61 |
| 3.30 | 5.67 | ENSG00000102531 | FNDC3A | Pr | Winter | 0 | 3.60E-01 | 3.30E-01 | 4.30E-01 | 2.28E-02 | 0.46 | 0.03 | -14.12 | -0.58 |
| 3.28 | 5.63 | ENSG00000188730 | VWC2 | rH | Summer | 0 | 4.90E-02 | 1.50E-01 | 1.80E-01 | 9.20E-01 | 1.00 | 0.16 | -13.45 | -0.71 |
| 3.28 | 5.62 | ENSG00000135973 | GPR45 | Pr | Summer | 0 | 6.60E-01 | 3.94E-02 | 1.30E-01 | 2.19E-02 | 0.50 | 0.04 | -1.87 | -0.26 |
| 3.28 | 5.62 | ENSG00000223220 | #NA# | Pr | Summer | 0 | 9.70E-01 | 6.80E-01 | 5.00E-02 | 4.44E-03 | 0.51 | 0.34 | -13.26 | -0.60 |
| 3.27 | 5.60 | ENSG00000158966 | CACHD1 | Pr | Winter | 0 | 3.50E-01 | 4.90E-01 | 2.80E-01 | 2.00E-01 | 0.57 | 0.03 | -2.28 | -0.17 |
| 3.27 | 5.60 | ENSG00000108819 | PPP1R9B | rH | Summer | 0 | 1.91E-02 | 1.50E-01 | 1.30E-01 | 6.00E-02 | 0.44 | 0.21 | -5.25 | -0.45 |
| 3.26 | 5.58 | ENSG00000153287 | #NA# | T | Winter | 0 | 2.12E-03 | 1.30E-01 | 1.40E-01 | 5.00E-02 | 0.49 | 0.31 | 4.45 | 0.40 |
| 3.26 | 5.58 | ENSG00000165795 | NDRG2 | Pr | Summer | 0 | 1.80E-01 | 7.20E-01 | 5.00E-02 | 1.71E-04 | 0.47 | 0.62 | -8.32 | -0.69 |
| 3.25 | 5.56 | ENSG00000066422 | ZBTB11 | T | Winter | 0 | 4.20E-01 | 1.20E-01 | 1.50E-01 | 5.25E-03 | 0.54 | 0.96 | -10.89 | -0.58 |
| 3.25 | 5.56 | ENSG00000054938 | CHRDL2 | T | Winter | 0 | 1.43E-02 | 4.00E-01 | 3.20E-01 | 4.35E-02 | 0.45 | 0.51 | -4.80 | -0.46 |
| 3.25 | 5.56 | ENSG00000174166 | #NA# | T | Winter | 0 | 6.10E-01 | 1.40E-01 | 2.50E-01 | 7.72E-04 | 0.53 | 0.72 | -8.41 | -0.51 |
| 3.25 | 5.56 | ENSG00000166404 | #NA# | Pr | Winter | 0 | 6.60E-01 | 1.00E-01 | 5.00E-02 | 1.40E-01 | 0.52 | 0.03 | -0.10 | 0.00 |
| 3.24 | 5.55 | ENSG00000158062 | UBXN11 | Pr | Summer | 0 | 9.80E-01 | 4.00E-01 | 2.60E-01 | 4.10E-05 | 0.45 | 0.68 | 0.20 | 0.01 |
| 3.24 | 5.54 | ENSG00000186113 | OR5D14 | Sr | Winter | 0 | 3.73E-02 | 5.50E-01 | 6.21E-03 | 1.31E-03 | 1.00 | 0.79 | -4.74 | -0.50 |
| 3.23 | 5.52 | ENSG00000222942 | #NA# | rH | Summer | 0 | 2.34E-03 | 7.00E-02 | 1.90E-01 | 1.10E-01 | 0.47 | 0.15 | -12.20 | -0.56 |
| 3.23 | 5.52 | ENSG00000203663 | OR2L2 | T | Winter | 0 | 2.72E-03 | 9.30E-01 | 5.60E-01 | 3.42E-03 | 0.56 | 0.20 | -4.77 | -0.19 |
| 3.22 | 5.51 | ENSG00000211277 | #NA# | Pr | Winter | 0 | 5.50E-01 | 3.00E-01 | 1.80E-01 | 1.10E-01 | 0.56 | 0.15 | -9.99 | -0.67 |
| 3.20 | 5.47 | ENSG00000172037 | LAMB2 | Sr | Winter | 0 | 1.70E-01 | 5.60E-01 | 1.20E-01 | 1.88E-04 | 0.55 | 0.66 | -16.07 | -0.56 |
| 3.20 | 5.47 | ENSG00000219751 | #NA# | rH | Summer | 0 | 1.20E-01 | 6.30E-01 | 6.00E-02 | 3.52E-02 | 1.00 | 0.25 | -21.26 | -0.79 |
| 3.20 | 5.46 | ENSG00000198053 | SIRPA | Pr | Summer | 0 | 8.30E-01 | 5.40E-01 | 1.90E-01 | 1.46E-04 | 1.00 | 0.16 | -3.77 | -0.33 |
| 3.20 | 5.46 | ENSG00000199788 | RNY3P2 | Pr | Winter | 0 | 1.60E-01 | 2.60E-01 | 6.70E-01 | 6.00E-02 | 0.51 | 0.09 | -13.96 | -0.61 |
| 3.18 | 5.42 | ENSG00000221124 | #NA# | Pr | Summer | 0 | 9.00E-01 | 2.80E-01 | 1.40E-01 | 1.58E-03 | 0.48 | 0.30 | 1.01 | 0.09 |
| 3.18 | 5.42 | ENSG00000201368 | #NA# | Pr | Winter | 0 | 8.00E-01 | 5.80E-01 | 3.10E-01 | 2.76E-04 | 1.00 | 0.33 | 1.70 | 0.08 |
| 3.17 | 5.41 | ENSG00000124568 | SLC17A1 | Pr | Winter | 0 | 5.20E-01 | 7.49E-03 | 1.21E-02 | 1.25E-02 | 1.00 | 0.11 | -7.70 | -0.55 |
| 3.17 | 5.40 | ENSG00000223202 | #NA# | T | Winter | 0 | 1.10E-02 | 3.40E-01 | 3.80E-01 | 5.56E-03 | 0.55 | 0.56 | -2.63 | -0.27 |
| 3.16 | 5.38 | ENSG00000172752 | COL6A5 | Pr | Summer | 0 | 9.10E-01 | 6.50E-01 | 3.20E-01 | 5.50E-05 | 0.44 | 0.21 | 4.68 | 0.31 |
| 3.14 | 5.34 | ENSG00000119042 | SATB2 | Pr | Summer | 0 | 6.90E-01 | 5.70E-01 | 1.10E-01 | 4.39E-03 | 0.49 | 0.03 | 0.13 | 0.01 |
| 3.14 | 5.34 | ENSG00000215864 | NBPF7 | Pr | Summer | 0 | 4.10E-01 | 9.50E-01 | 2.20E-01 | 1.18E-04 | 0.53 | 0.19 | 4.51 | 0.30 |
| 3.14 | 5.34 | ENSG00000174021 | GNG5 | T | Winter | 0 | 3.30E-01 | 9.70E-01 | 9.03E-03 | 2.13E-03 | 1.00 | 0.10 | 2.34 | 0.13 |
| 3.14 | 5.34 | ENSG00000218509 | #NA# | rH | Summer | 0 | 6.40E-01 | 1.73E-02 | 2.67E-02 | 8.91E-03 | 0.52 | 0.32 | -10.80 | -0.55 |
| 3.13 | 5.33 | ENSG00000176273 | SLC35G1 | rH | Summer | 0 | 6.00E-02 | 2.36E-02 | 7.00E-02 | 1.40E-01 | 0.44 | 0.42 | -11.09 | -0.49 |
| 3.11 | 5.29 | ENSG00000142611 | PRDM16 | Sr | Winter | 0 | 8.67E-04 | 8.70E-01 | 3.40E-01 | 8.00E-02 | 0.48 | 0.95 | 1.08 | 0.07 |
| 3.11 | 5.28 | ENSG00000164434 | FABP7 | Pr | Summer | 0 | 9.60E-01 | 3.90E-01 | 3.50E-01 | 2.34E-03 | 1.00 | 0.12 | -4.79 | -0.37 |
| 3.11 | 5.28 | ENSG00000207056 | #NA# | rH | Summer | 0 | 1.96E-02 | 1.90E-01 | 3.13E-02 | 1.50E-01 | 1.00 | 0.41 | -8.84 | -0.38 |
| 3.10 | 5.27 | ENSG00000217689 | #NA# | Sr | Winter | 0 | 3.50E-01 | 7.00E-02 | 8.00E-02 | 1.50E-01 | 0.51 | 0.13 | -5.68 | -0.41 |
| 3.10 | 5.26 | ENSG00000106571 | GLI3 | T | Winter | 0 | 6.73E-04 | 2.80E-01 | 4.42E-02 | 7.00E-02 | 1.00 | 0.27 | -4.75 | -0.32 |
| 3.10 | 5.26 | ENSG00000198877 | OR5D13 | Sr | Winter | 0 | 4.46E-02 | 3.80E-01 | 7.07E-03 | 1.43E-03 | 1.00 | 0.92 | -6.29 | -0.49 |
| 3.10 | 5.26 | ENSG00000222463 | #NA# | Pr | Winter | 0 | 9.50E-01 | 4.40E-01 | 1.60E-01 | 1.20E-01 | 0.50 | 0.04 | 2.50 | 0.14 |
| 3.09 | 5.25 | ENSG00000213704 | EEF1A1P15 | T | Winter | 0 | 5.00E-02 | 2.40E-01 | 2.28E-02 | 2.09E-03 | 1.00 | 0.09 | 1.32 | 0.10 |
| 3.09 | 5.25 | ENSG00000173575 | CHD2 | rH | Summer | 0 | 6.00E-02 | 1.30E-01 | 1.50E-01 | 1.40E-01 | 1.00 | 0.16 | -8.31 | -0.53 |
| 3.08 | 5.22 | ENSG00000159214 | CCDC24 | T | Winter | 0 | 5.00E-02 | 7.80E-01 | 3.10E-01 | 1.31E-02 | 0.50 | 0.12 | 4.22 | 0.28 |
| 3.07 | 5.21 | ENSG00000218501 | #NA# | rH | Summer | 0 | 3.29E-02 | 6.00E-02 | 2.50E-01 | 2.90E-01 | 0.50 | 0.11 | -18.98 | -0.78 |
| 3.07 | 5.20 | ENSG00000170871 | KIAA0232 | Pr | Winter | 0 | 7.40E-01 | 1.90E-01 | 1.70E-01 | 2.11E-02 | 1.00 | 0.14 | 0.96 | 0.07 |
| 3.07 | 5.20 | ENSG00000199585 | RN5S119 | rH | Summer | 0 | 9.23E-03 | 1.40E-01 | 4.90E-02 | 1.20E-01 | 0.41 | 0.96 | -8.64 | -0.56 |
| 3.06 | 5.19 | ENSG00000167641 | PPP1R14A | Pr | Winter | 0 | 9.60E-01 | 3.10E-01 | 1.40E-02 | 1.30E-01 | 1.00 | 0.06 | -3.66 | -0.29 |
| 3.06 | 5.19 | ENSG00000208783 | #NA# | rH | Summer | 0 | 8.67E-03 | 6.80E-01 | 7.00E-02 | 2.67E-02 | 0.59 | 0.21 | -2.95 | -0.26 |
| 3.06 | 5.19 | ENSG00000175906 | ARL4D | Pr | Winter | 0 | 7.20E-01 | 4.80E-01 | 4.10E-01 | 1.98E-02 | 0.55 | 0.54 | -9.92 | -0.55 |
| 3.06 | 5.19 | ENSG00000113407 | TARS | Pr | Winter | 0 | 6.00E-01 | 3.40E-01 | 5.00E-02 | 2.37E-02 | 0.48 | 0.04 | -2.39 | -0.22 |
| 3.06 | 5.18 | ENSG00000209662 | #NA# | rH | Summer | 0 | 2.78E-02 | 1.60E-01 | 2.20E-01 | 5.60E-05 | 0.46 | 0.70 | -1.90 | -0.17 |
| 3.05 | 5.17 | ENSG00000200550 | #NA# | Sr | Winter | 0 | 5.00E-02 | 7.90E-01 | 1.80E-01 | 2.55E-02 | 0.48 | 0.41 | -7.83 | -0.42 |
| 3.05 | 5.16 | ENSG00000213002 | #NA# | Pr | Summer | 0 | 9.10E-01 | 9.80E-01 | 1.10E-01 | 1.00E-02 | 1.00 | 0.17 | -22.06 | -0.57 |
| 3.04 | 5.16 | ENSG00000220611 | #NA# | Pr | Winter | 0 | 9.00E-02 | 1.90E-01 | 7.00E-02 | 2.20E-01 | 0.56 | 0.44 | -13.30 | -0.58 |
| 3.04 | 5.15 | ENSG00000175866 | BAIAP2 | Sr | Winter | 0 | 5.70E-01 | 8.00E-01 | 3.47E-02 | 7.99E-04 | 0.58 | 0.55 | -3.30 | -0.40 |
| 3.04 | 5.15 | ENSG00000149679 | CABLES2 | rH | Summer | 0 | 1.58E-02 | 1.30E-01 | 7.80E-01 | 1.32E-02 | 1.00 | 0.73 | -10.62 | -0.66 |
| 3.04 | 5.14 | ENSG00000168958 | MFF | T | Winter | 0 | 9.00E-02 | 6.60E-01 | 1.70E-01 | 5.19E-03 | 0.61 | 0.30 | -4.29 | -0.33 |
| 3.03 | 5.13 | ENSG00000201965 | #NA# | rH | Summer | 0 | 4.03E-02 | 8.00E-02 | 8.00E-02 | 2.00E-01 | 0.53 | 0.63 | -5.74 | -0.43 |
| 3.03 | 5.13 | ENSG00000125881 | #NA# | T | Winter | 0 | 4.63E-02 | 5.50E-01 | 2.20E-01 | 9.37E-04 | 1.00 | 0.06 | 0.40 | 0.02 |
| 3.03 | 5.12 | ENSG00000210583 | #NA# | Pr | Winter | 0 | 4.13E-02 | 4.30E-01 | 1.68E-02 | 2.17E-03 | 0.50 | 0.04 | -6.00 | -0.63 |
| 3.03 | 5.12 | ENSG00000186642 | PDE2A | Pr | Summer | 0 | 4.10E-01 | 3.60E-01 | 6.00E-02 | 3.14E-03 | 1.00 | 0.20 | -11.66 | -0.72 |
| 3.03 | 5.12 | ENSG00000213247 | #NA# | Pr | Summer | 0 | 3.60E-01 | 5.10E-01 | 4.81E-02 | 7.69E-04 | 1.00 | 0.52 | 2.03 | 0.16 |
| 3.02 | 5.11 | ENSG00000209723 | #NA# | Pr | Summer | 0 | 7.73E-03 | 9.00E-02 | 4.46E-02 | 2.57E-03 | 0.53 | 0.14 | 0.64 | 0.07 |
| 3.02 | 5.11 | ENSG00000181961 | OR4A16 | Sr | Winter | 0 | 2.31E-02 | 3.80E-01 | 4.46E-02 | 1.70E-03 | 1.00 | 0.70 | -6.72 | -0.39 |
| 3.02 | 5.10 | ENSG00000197430 | OPALIN | Pr | Summer | 0 | 6.50E-01 | 1.70E-01 | 1.20E-01 | 4.92E-03 | 0.49 | 0.21 | -0.69 | -0.05 |
| 3.02 | 5.10 | ENSG00000203710 | CR1 | Sr | Winter | 0 | 2.40E-01 | 7.10E-01 | 6.95E-03 | 9.88E-03 | 1.00 | 0.02 | -1.80 | -0.15 |
| 3.02 | 5.10 | ENSG00000209417 | #NA# | Pr | Winter | 0 | 7.90E-01 | 1.70E-01 | 5.00E-02 | 4.10E-02 | 0.65 | 0.38 | -7.62 | -0.57 |
| 3.01 | 5.09 | ENSG00000130035 | GALNT8 | Sr | Winter | 0 | 2.30E-01 | 3.00E-01 | 8.00E-02 | 6.06E-03 | 0.49 | 0.47 | 0.35 | 0.03 |
| 3.01 | 5.09 | ENSG00000200982 | #NA# | T | Winter | 0 | 1.36E-02 | 9.90E-01 | 7.70E-01 | 1.19E-03 | 0.50 | 0.18 | -6.00 | -0.24 |
| 2.99 | 5.04 | ENSG00000187260 | WDR86 | T | Summer | 0 | 2.20E-03 | 5.70E-01 | 2.00E-01 | 1.55E-02 | 1.00 | 0.82 | -4.89 | -0.38 |
| 2.98 | 5.03 | ENSG00000198179 | #NA# | Pr | Winter | 0 | 9.70E-01 | 4.90E-01 | 5.40E-01 | 8.00E-02 | 1.00 | 0.52 | -17.92 | -0.69 |
| 2.98 | 5.02 | ENSG00000197067 | #NA# | T | Winter | 0 | 1.60E-01 | 9.90E-01 | 5.80E-01 | 4.24E-04 | 0.48 | 0.21 | -4.57 | -0.22 |
| 2.98 | 5.02 | ENSG00000218629 | #NA# | rH | Summer | 0 | 8.00E-02 | 1.10E-01 | 3.33E-02 | 2.56E-02 | 1.00 | 0.57 | -15.44 | -0.80 |
| 2.98 | 5.02 | ENSG00000184774 | #NA# | Pr | Summer | 0 | 8.00E-02 | 3.70E-01 | 7.00E-02 | 6.34E-03 | 0.44 | 0.81 | -1.01 | -0.08 |
| 2.98 | 5.02 | ENSG00000178084 | HTR3C | Pr | Winter | 0 | 4.60E-01 | 5.30E-01 | 4.30E-01 | 1.60E-02 | 0.48 | 0.10 | -5.54 | -0.46 |
| 2.97 | 5.01 | ENSG00000116991 | SIPA1L2 | Pr | Winter | 0 | 2.30E-01 | 1.40E-01 | 1.01E-02 | 1.53E-02 | 0.53 | 0.22 | -4.09 | -0.27 |
| 2.97 | 5.01 | ENSG00000219703 | #NA# | Sr | Winter | 0 | 2.20E-03 | 1.40E-01 | 2.34E-02 | 4.31E-02 | 0.45 | 0.44 | -6.06 | -0.49 |
| 2.97 | 5.01 | ENSG00000203711 | C6orf99 | Pr | Winter | 0 | 1.00E+00 | 5.30E-01 | 5.00E-02 | 2.30E-02 | 0.50 | 0.26 | -14.07 | -0.79 |
| 2.96 | 4.99 | ENSG00000223169 | #NA# | Pr | Winter | 0 | 3.80E-01 | 5.00E-01 | 1.90E-01 | 1.10E-01 | 0.43 | 0.25 | -10.21 | -0.68 |
| 2.96 | 4.98 | ENSG00000210048 | #NA# | rH | Summer | 0 | 2.49E-02 | 3.65E-02 | 3.10E-01 | 3.30E-01 | 0.48 | 0.20 | -3.42 | -0.37 |
| 2.95 | 4.97 | ENSG00000213247 | #NA# | T | Winter | 0 | 2.49E-02 | 3.40E-01 | 3.42E-02 | 1.63E-02 | 1.00 | 0.62 | 2.03 | 0.16 |
| 2.95 | 4.97 | ENSG00000162139 | NEU3 | Pr | Winter | 0 | 4.10E-01 | 6.10E-01 | 5.00E-01 | 3.36E-02 | 1.00 | 0.09 | -4.10 | -0.28 |
| 2.95 | 4.96 | ENSG00000077514 | POLD3 | T | Winter | 0 | 5.00E-01 | 3.80E-01 | 1.00E-01 | 2.97E-03 | 1.00 | 0.57 | -2.39 | -0.18 |
| 2.94 | 4.95 | ENSG00000220563 | #NA# | Sr | Winter | 0 | 1.25E-02 | 9.50E-01 | 6.00E-02 | 5.00E-02 | 0.53 | 0.60 | -11.65 | -0.60 |
| 2.93 | 4.93 | ENSG00000219302 | #NA# | Pr | Winter | 0 | 8.90E-01 | 6.90E-01 | 6.30E-01 | 5.53E-03 | 0.54 | 0.16 | -7.69 | -0.57 |
| 2.93 | 4.93 | ENSG00000158486 | DNAH3 | T | Winter | 0 | 1.00E-01 | 4.60E-01 | 2.00E-01 | 5.40E-05 | 0.51 | 0.09 | 3.65 | 0.24 |
| 2.93 | 4.92 | ENSG00000178537 | SLC25A20 | Sr | Winter | 0 | 7.00E-02 | 6.60E-01 | 1.10E-01 | 1.18E-03 | 0.42 | 0.82 | -20.84 | -0.59 |
| 2.93 | 4.92 | ENSG00000186832 | KRT16 | rH | Summer | 0 | 1.81E-02 | 2.60E-01 | 1.70E-01 | 7.00E-02 | 1.00 | 0.69 | -20.72 | -0.72 |
| 2.92 | 4.91 | ENSG00000180715 | #NA# | rH | Summer | 0 | 3.64E-04 | 7.97E-03 | 2.00E-01 | 5.80E-01 | 0.49 | 0.58 | -10.42 | -0.70 |
| 2.92 | 4.90 | ENSG00000218766 | #NA# | Sr | Winter | 0 | 5.54E-03 | 9.90E-01 | 4.76E-02 | 3.40E-02 | 1.00 | 0.06 | -9.70 | -0.41 |
| 2.91 | 4.89 | ENSG00000212167 | #NA# | Sr | Winter | 0 | 1.81E-02 | 7.80E-01 | 2.50E-01 | 2.06E-03 | 0.47 | 0.50 | -0.22 | -0.02 |
| 2.91 | 4.88 | ENSG00000200085 | #NA# | T | Winter | 0 | 2.34E-03 | 9.70E-01 | 6.40E-01 | 1.27E-03 | 0.52 | 0.19 | -9.48 | -0.31 |
| 2.90 | 4.87 | ENSG00000206981 | #NA# | Pr | Summer | 0 | 8.90E-01 | 9.00E-02 | 1.30E-01 | 9.79E-03 | 0.45 | 0.71 | -7.45 | -0.48 |
| 2.90 | 4.86 | ENSG00000186113 | OR5D14 | T | Winter | 0 | 6.00E-02 | 3.00E-01 | 6.00E-02 | 5.27E-03 | 0.56 | 0.81 | -4.74 | -0.50 |
| 2.89 | 4.85 | ENSG00000211499 | #NA# | Pr | Winter | 0 | 6.10E-01 | 1.10E-01 | 3.40E-01 | 3.45E-02 | 1.00 | 0.07 | -6.81 | -0.54 |
| 2.89 | 4.85 | ENSG00000209362 | #NA# | rH | Winter | 0 | 8.93E-03 | 7.30E-01 | 2.20E-01 | 2.25E-03 | 0.58 | 0.07 | -2.95 | -0.22 |
| 2.88 | 4.83 | ENSG00000221955 | SLC12A8 | Sr | Winter | 0 | 1.30E-01 | 8.50E-01 | 6.46E-03 | 7.00E-05 | 0.46 | 0.85 | -0.02 | 0.00 |
| 2.87 | 4.80 | ENSG00000200830 | #NA# | Sr | Winter | 0 | 6.00E-02 | 3.20E-01 | 3.60E-01 | 4.42E-02 | 0.56 | 0.36 | -4.96 | -0.45 |
| 2.86 | 4.78 | ENSG00000212249 | #NA# | Pr | Winter | 0 | 9.60E-01 | 1.40E-01 | 3.30E-01 | 1.85E-02 | 1.00 | 0.04 | -8.29 | -0.47 |
| 2.86 | 4.78 | ENSG00000199260 | #NA# | Sr | Winter | 0 | 8.00E-02 | 8.20E-01 | 1.93E-02 | 1.00E-01 | 0.51 | 0.46 | -6.57 | -0.56 |
| 2.85 | 4.76 | ENSG00000172890 | NADSYN1 | Sr | Winter | 0 | 5.00E-01 | 5.90E-01 | 1.46E-02 | 1.18E-03 | 1.00 | 0.49 | 1.71 | 0.11 |
| 2.84 | 4.75 | ENSG00000139209 | SLC38A4 | Pr | Winter | 0 | 9.70E-01 | 5.30E-01 | 1.00E-01 | 2.60E-02 | 0.62 | 0.19 | -0.64 | -0.06 |
| 2.84 | 4.75 | ENSG00000219277 | #NA# | T | Winter | 0 | 1.45E-03 | 9.60E-01 | 7.70E-01 | 3.46E-03 | 0.59 | 0.17 | -6.76 | -0.33 |
| 2.84 | 4.74 | ENSG00000215899 | #NA# | Pr | Summer | 0 | 9.40E-01 | 1.40E-01 | 2.30E-01 | 7.83E-03 | 0.49 | 0.62 | -8.36 | -0.60 |
| 2.84 | 4.74 | ENSG00000138463 | DIRC2 | Pr | Winter | 0 | 8.50E-01 | 4.50E-01 | 8.77E-03 | 1.23E-02 | 1.00 | 0.23 | -7.64 | -0.50 |
| 2.83 | 4.72 | ENSG00000212153 | #NA# | Pr | Winter | 0 | 5.90E-01 | 2.39E-02 | 2.50E-01 | 6.00E-02 | 0.50 | 0.28 | -7.11 | -0.37 |
| 2.82 | 4.71 | ENSG00000150048 | CLEC1A | Pr | Summer | 0 | 8.50E-01 | 5.10E-01 | 4.24E-02 | 1.75E-02 | 0.51 | 0.44 | -6.27 | -0.33 |
| 2.82 | 4.71 | ENSG00000211277 | #NA# | Pr | Summer | 0 | 2.40E-01 | 2.40E-01 | 3.00E-01 | 6.00E-02 | 0.45 | 0.05 | -9.99 | -0.67 |
| 2.82 | 4.70 | ENSG00000166007 | #NA# | Sr | Winter | 0 | 1.60E-01 | 5.80E-01 | 8.00E-02 | 2.49E-03 | 1.00 | 0.97 | -12.74 | -0.49 |
| 2.81 | 4.70 | ENSG00000203791 | METTL10 | T | Winter | 0 | 8.50E-01 | 3.00E-01 | 2.14E-02 | 9.86E-03 | 0.50 | 0.49 | -9.01 | -0.38 |
| 2.81 | 4.69 | ENSG00000082556 | OPRK1 | Sr | Winter | 0 | 1.27E-03 | 6.40E-01 | 8.00E-02 | 4.50E-01 | 0.42 | 0.45 | -4.80 | -0.36 |
| 2.80 | 4.67 | ENSG00000204646 | #NA# | Pr | Summer | 0 | 2.20E-01 | 4.30E-01 | 3.52E-02 | 1.84E-03 | 1.00 | 0.30 | -7.83 | -0.60 |
| 2.80 | 4.66 | ENSG00000181104 | F2R | T | Winter | 0 | 7.00E-01 | 4.90E-01 | 2.90E-01 | 7.00E-03 | 1.00 | 0.11 | -5.43 | -0.42 |
| 2.79 | 4.65 | ENSG00000130158 | DOCK6 | T | Summer | 0 | 1.10E-01 | 3.10E-01 | 1.01E-02 | 2.78E-03 | 0.47 | 0.34 | 11.89 | 0.63 |
| 2.79 | 4.64 | ENSG00000085832 | EPS15 | Sr | Summer | 0 | 4.46E-02 | 8.00E-02 | 1.50E-01 | 6.00E-02 | 0.42 | 0.71 | 2.99 | 0.18 |
| 2.77 | 4.61 | ENSG00000201149 | #NA# | T | Winter | 0 | 6.00E-02 | 4.50E-01 | 1.60E-01 | 3.31E-02 | 1.00 | 0.18 | -7.51 | -0.50 |
| 2.77 | 4.60 | ENSG00000139209 | SLC38A4 | Pr | Summer | 0 | 3.80E-01 | 6.00E-02 | 1.10E-01 | 2.06E-03 | 1.00 | 0.39 | -0.64 | -0.06 |
| 2.76 | 4.59 | ENSG00000218479 | #NA# | Pr | Winter | 0 | 6.80E-01 | 4.10E-01 | 6.00E-01 | 5.00E-02 | 0.47 | 0.03 | -15.55 | -0.71 |
| 2.75 | 4.57 | ENSG00000130035 | GALNT8 | T | Winter | 0 | 3.10E-01 | 2.80E-01 | 2.90E-01 | 3.99E-03 | 1.00 | 0.16 | 0.35 | 0.03 |
| 2.75 | 4.57 | ENSG00000197763 | TXNRD3 | T | Winter | 0 | 1.10E-01 | 7.60E-01 | 8.00E-02 | 6.86E-03 | 1.00 | 0.25 | -2.63 | -0.18 |
| 2.75 | 4.56 | ENSG00000198077 | CYP2A7 | T | Winter | 0 | 1.80E-01 | 4.30E-01 | 5.50E-01 | 1.51E-03 | 1.00 | 0.11 | -10.33 | -0.62 |
| 2.74 | 4.56 | ENSG00000171105 | INSR | Sr | Winter | 0 | 1.00E-01 | 2.50E-01 | 1.40E-01 | 5.54E-03 | 0.48 | 0.60 | -2.26 | -0.18 |
| 2.74 | 4.55 | ENSG00000207296 | #NA# | Pr | Winter | 0 | 1.00E+00 | 7.00E-01 | 3.10E-01 | 6.00E-02 | 1.00 | 0.10 | -4.68 | -0.30 |
| 2.74 | 4.55 | ENSG00000217017 | #NA# | Pr | Winter | 0 | 7.80E-01 | 4.67E-02 | 9.00E-02 | 3.30E-01 | 1.00 | 0.05 | -7.23 | -0.34 |
| 2.74 | 4.54 | ENSG00000131941 | RHPN2 | Sr | Winter | 0 | 1.70E-01 | 3.90E-01 | 3.45E-02 | 1.00E-01 | 0.41 | 0.47 | -3.93 | -0.32 |
| 2.73 | 4.53 | ENSG00000177752 | YIPF7 | Sr | Winter | 0 | 8.00E-02 | 2.20E-01 | 5.80E-01 | 2.79E-03 | 0.57 | 0.09 | -1.00 | -0.10 |
| 2.73 | 4.53 | ENSG00000160220 | #NA# | T | Winter | 0 | 2.91E-02 | 2.60E-01 | 5.00E-02 | 1.22E-03 | 0.47 | 0.26 | 5.29 | 0.22 |
| 2.73 | 4.52 | ENSG00000196071 | OR2L13 | T | Winter | 0 | 6.05E-03 | 9.80E-01 | 8.30E-01 | 1.13E-02 | 0.50 | 0.29 | -5.95 | -0.35 |
| 2.72 | 4.51 | ENSG00000160221 | C21orf33 | Sr | Winter | 0 | 3.30E-01 | 5.10E-01 | 4.46E-02 | 3.65E-02 | 1.00 | 0.07 | 0.67 | 0.04 |
| 2.72 | 4.51 | ENSG00000209656 | #NA# | T | Winter | 0 | 1.98E-02 | 5.01E-03 | 6.10E-01 | 1.58E-03 | 0.48 | 0.99 | 2.06 | 0.09 |
| 2.71 | 4.49 | ENSG00000178934 | LGALS7B | Pr | Winter | 0 | 8.50E-01 | 7.00E-01 | 3.70E-01 | 6.00E-02 | 1.00 | 0.29 | -4.80 | -0.28 |
| 2.70 | 4.47 | ENSG00000110492 | MDK | Pr | Summer | 0 | 1.90E-01 | 3.20E-01 | 4.40E-01 | 2.78E-04 | 0.44 | 0.41 | -7.44 | -0.51 |
| 2.70 | 4.46 | ENSG00000100554 | ATP6V1D | Pr | Summer | 0 | 1.00E+00 | 5.60E-01 | 3.36E-02 | 4.76E-02 | 1.00 | 0.66 | 1.96 | 0.11 |
| 2.69 | 4.45 | ENSG00000082641 | NFE2L1 | rH | Summer | 0 | 4.17E-02 | 4.55E-02 | 4.10E-01 | 5.00E-02 | 0.48 | 0.38 | -6.61 | -0.40 |
| 2.68 | 4.44 | ENSG00000147654 | EBAG9 | T | Winter | 0 | 1.30E-01 | 6.50E-01 | 4.30E-01 | 7.14E-03 | 0.47 | 0.27 | 0.29 | 0.02 |
| 2.68 | 4.44 | ENSG00000222156 | #NA# | Pr | Summer | 0 | 7.80E-01 | 1.70E-01 | 9.00E-02 | 3.25E-02 | 0.51 | 0.04 | -8.11 | -0.47 |
| 2.68 | 4.44 | ENSG00000118514 | ALDH8A1 | Sr | Winter | 0 | 7.00E-02 | 1.90E-01 | 5.00E-02 | 1.76E-02 | 0.55 | 0.30 | 5.40 | 0.31 |
| 2.68 | 4.43 | ENSG00000145780 | FEM1C | rH | Summer | 0 | 2.89E-02 | 1.96E-02 | 4.85E-02 | 1.80E-01 | 1.00 | 0.59 | -9.56 | -0.61 |
| 2.68 | 4.43 | ENSG00000222704 | #NA# | T | Winter | 0 | 8.20E-01 | 8.40E-01 | 6.20E-01 | 1.88E-02 | 0.57 | 0.22 | -4.15 | -0.29 |
| 2.68 | 4.43 | ENSG00000135820 | #NA# | T | Winter | 0 | 1.50E-01 | 9.42E-03 | 7.60E-01 | 1.82E-02 | 0.48 | 0.16 | -0.58 | -0.05 |
| 2.67 | 4.40 | ENSG00000214688 | C10orf105 | Sr | Winter | 1 | 7.00E-02 | 2.40E-01 | 6.60E-01 | 0.00E+00 | 1.00 | 0.66 | -1.43 | -0.19 |
| 2.66 | 4.39 | ENSG00000121316 | PLBD1 | Pr | Winter | 0 | 5.70E-01 | 6.70E-01 | 2.10E-01 | 6.80E-01 | 1.00 | 0.05 | -1.79 | -0.22 |
| 2.65 | 4.37 | ENSG00000207963 | MIR569 | T | Winter | 0 | 6.10E-01 | 3.90E-04 | 1.04E-02 | 2.34E-02 | 0.52 | 0.20 | -3.91 | -0.44 |
| 2.64 | 4.35 | ENSG00000173334 | TRIB1 | Sr | Winter | 0 | 5.00E-02 | 4.90E-01 | 4.40E-01 | 1.06E-02 | 1.00 | 0.27 | -3.27 | -0.37 |
| 2.63 | 4.33 | ENSG00000143994 | ABHD1 | Pr | Winter | 0 | 2.00E-01 | 1.50E-01 | 1.50E-01 | 5.00E-01 | 0.48 | 0.18 | -5.19 | -0.23 |
| 2.61 | 4.30 | ENSG00000144741 | SLC25A26 | Pr | Summer | 0 | 4.90E-01 | 8.00E-02 | 1.70E-01 | 3.36E-02 | 0.50 | 0.02 | -8.61 | -0.48 |
| 2.61 | 4.29 | ENSG00000163600 | ICOS | T | Winter | 0 | 4.07E-02 | 4.60E-01 | 6.00E-02 | 6.86E-03 | 0.50 | 0.05 | -0.68 | -0.06 |
| 2.61 | 4.28 | ENSG00000112208 | BAG2 | Sr | Summer | 0 | 2.86E-02 | 1.90E-01 | 7.20E-01 | 3.80E-05 | 0.42 | 0.33 | -10.79 | -0.59 |
| 2.61 | 4.28 | ENSG00000213658 | LAT | Sr | Winter | 0 | 5.10E-03 | 3.03E-02 | 4.81E-02 | 9.19E-03 | 0.54 | 0.77 | -1.53 | -0.12 |
| 2.60 | 4.27 | ENSG00000146109 | ABT1 | Sr | Winter | 0 | 5.00E-02 | 1.45E-02 | 3.13E-02 | 9.55E-04 | 0.54 | 0.89 | -4.67 | -0.37 |
| 2.59 | 4.25 | ENSG00000081154 | PCNP | T | Winter | 0 | 1.10E-01 | 5.80E-01 | 2.30E-01 | 2.49E-03 | 0.50 | 0.63 | -5.66 | -0.28 |
| 2.59 | 4.25 | ENSG00000204178 | TMEM57 | Pr | Summer | 0 | 8.90E-01 | 6.70E-01 | 5.10E-01 | 4.88E-03 | 0.46 | 0.15 | -6.83 | -0.31 |
| 2.56 | 4.19 | ENSG00000115486 | GGCX | T | Winter | 0 | 5.70E-01 | 2.58E-03 | 2.40E-01 | 1.89E-03 | 1.00 | 1.00 | -11.85 | -0.57 |
| 2.56 | 4.19 | ENSG00000142669 | SH3BGRL3 | Pr | Winter | 0 | 5.50E-01 | 7.00E-02 | 1.40E-01 | 3.11E-03 | 0.45 | 0.04 | -11.70 | -0.61 |
| 2.55 | 4.17 | ENSG00000218054 | #NA# | rH | Summer | 0 | 1.56E-03 | 3.20E-01 | 5.20E-01 | 6.00E-02 | 1.00 | 0.84 | -11.29 | -0.65 |
| 2.54 | 4.15 | ENSG00000060971 | ACAA1 | rH | Summer | 0 | 1.40E-01 | 3.91E-02 | 2.49E-02 | 6.20E-01 | 0.60 | 0.60 | -4.92 | -0.36 |
| 2.54 | 4.15 | ENSG00000213370 | RANP6 | T | Winter | 0 | 6.00E-02 | 4.90E-01 | 3.40E-01 | 1.90E-01 | 0.38 | 0.10 | -9.72 | -0.65 |
| 2.54 | 4.14 | ENSG00000026508 | CD44 | T | Winter | 0 | 9.38E-04 | 2.91E-02 | 3.10E-01 | 1.40E-01 | 0.53 | 0.38 | -5.48 | -0.51 |
| 2.54 | 4.14 | ENSG00000091010 | POU4F3 | T | Winter | 0 | 7.70E-01 | 6.10E-01 | 4.20E-01 | 4.20E-02 | 0.51 | 0.13 | -6.16 | -0.32 |
| 2.53 | 4.14 | ENSG00000201314 | #NA# | Sr | Winter | 0 | 2.25E-02 | 4.20E-01 | 3.27E-02 | 8.21E-04 | 0.53 | 0.60 | -5.39 | -0.51 |
| 2.53 | 4.13 | ENSG00000221103 | #NA# | Sr | Winter | 0 | 3.10E-01 | 5.10E-01 | 2.50E-01 | 1.49E-02 | 0.48 | 0.54 | -2.91 | -0.18 |
| 2.51 | 4.09 | ENSG00000203729 | LINC00272 | T | Winter | 0 | 7.30E-01 | 1.90E-01 | 1.70E-01 | 2.10E-03 | 1.00 | 0.04 | -0.84 | -0.07 |
| 2.51 | 4.08 | ENSG00000135898 | GPR55 | Sr | Winter | 0 | 1.40E-01 | 4.50E-01 | 9.00E-02 | 2.59E-02 | 0.48 | 0.10 | -3.86 | -0.26 |
| 2.50 | 4.08 | ENSG00000143184 | XCL1 | Pr | Summer | 0 | 1.00E+00 | 7.00E-01 | 4.20E-01 | 2.70E-05 | 1.00 | 0.09 | -9.64 | -0.57 |
| 2.50 | 4.06 | ENSG00000211733 | #NA# | rH | Summer | 0 | 6.52E-03 | 6.00E-02 | 4.90E-01 | 8.48E-03 | 0.50 | 0.82 | -5.08 | -0.45 |
| 2.49 | 4.04 | ENSG00000112210 | RAB23 | Sr | Summer | 0 | 1.28E-02 | 2.10E-01 | 6.20E-01 | 1.93E-04 | 0.51 | 0.69 | -10.51 | -0.57 |
| 2.49 | 4.04 | ENSG00000169575 | VPREB1 | Sr | Winter | 0 | 4.30E-01 | 9.70E-01 | 1.50E-01 | 7.51E-03 | 0.50 | 0.16 | -2.80 | -0.20 |
| 2.47 | 4.00 | ENSG00000160218 | TRAPPC10 | Sr | Winter | 0 | 5.60E-01 | 5.50E-01 | 8.00E-02 | 6.00E-02 | 1.00 | 0.34 | 2.90 | 0.17 |
| 2.47 | 4.00 | ENSG00000160013 | PTGIR | rH | Summer | 0 | 6.10E-01 | 5.68E-03 | 3.50E-01 | 5.16E-03 | 0.51 | 0.16 | -14.45 | -0.74 |
| 2.47 | 4.00 | ENSG00000201511 | #NA# | T | Winter | 0 | 2.60E-01 | 1.70E-01 | 2.10E-01 | 4.27E-03 | 0.62 | 0.95 | -11.99 | -0.58 |
| 2.45 | 3.96 | ENSG00000213721 | HMGN2P30 | T | Winter | 0 | 3.01E-03 | 3.50E-01 | 3.62E-02 | 1.50E-01 | 1.00 | 0.27 | -0.34 | -0.02 |
| 2.44 | 3.95 | ENSG00000217334 | #NA# | T | Winter | 0 | 2.00E-01 | 9.50E-01 | 3.90E-01 | 1.40E-01 | 1.00 | 0.74 | -18.28 | -0.51 |
| 2.42 | 3.91 | ENSG00000199466 | #NA# | T | Winter | 0 | 8.80E-01 | 4.60E-01 | 2.70E-02 | 4.03E-02 | 0.41 | 0.60 | -10.95 | -0.45 |
| 2.41 | 3.89 | ENSG00000107447 | DNTT | Pr | Winter | 0 | 8.60E-01 | 6.10E-01 | 5.40E-01 | 2.72E-03 | 0.51 | 0.07 | -0.94 | -0.05 |
| 2.41 | 3.89 | ENSG00000182504 | CEP97 | T | Winter | 0 | 5.00E-01 | 1.80E-01 | 3.70E-01 | 1.66E-03 | 0.49 | 0.91 | -6.40 | -0.44 |
| 2.40 | 3.87 | ENSG00000105700 | C19orf50 | Sr | Summer | 0 | 4.05E-04 | 7.00E-02 | 3.00E-01 | 4.63E-02 | 0.57 | 1.00 | -10.39 | -0.44 |
| 2.40 | 3.87 | ENSG00000104885 | DOT1L | T | Winter | 0 | 3.91E-03 | 3.40E-01 | 3.10E-01 | 2.03E-02 | 0.44 | 0.10 | -9.53 | -0.54 |
| 2.39 | 3.86 | ENSG00000217203 | #NA# | Sr | Winter | 0 | 6.60E-01 | 4.10E-01 | 7.00E-02 | 6.00E-02 | 1.00 | 0.33 | 1.77 | 0.12 |
| 2.39 | 3.85 | ENSG00000183524 | #NA# | T | Winter | 0 | 6.00E-02 | 6.00E-01 | 2.40E-01 | 2.84E-03 | 0.53 | 0.65 | -5.11 | -0.26 |
| 2.38 | 3.83 | ENSG00000100413 | POLR3H | Pr | Summer | 0 | 1.60E-01 | 2.20E-01 | 3.80E-01 | 6.57E-04 | 0.50 | 0.78 | -8.03 | -0.43 |
| 2.36 | 3.78 | ENSG00000114316 | USP4 | Pr | Summer | 0 | 9.80E-01 | 8.00E-01 | 1.30E-02 | 2.75E-02 | 0.56 | 0.80 | -18.06 | -0.62 |
| 2.34 | 3.74 | ENSG00000173726 | TOMM20 | Pr | Summer | 0 | 4.90E-01 | 1.40E-01 | 3.90E-01 | 1.99E-04 | 0.54 | 0.49 | -6.48 | -0.44 |
| 2.31 | 3.69 | ENSG00000203710 | CR1 | T | Winter | 0 | 2.90E-01 | 8.80E-01 | 2.78E-02 | 1.36E-02 | 0.52 | 0.49 | -1.80 | -0.15 |
| 2.30 | 3.66 | ENSG00000169442 | CD52 | Pr | Summer | 0 | 9.90E-01 | 4.80E-01 | 4.00E-01 | 3.10E-05 | 0.57 | 0.76 | -3.38 | -0.18 |
| 2.29 | 3.66 | ENSG00000202069 | #NA# | T | Winter | 0 | 1.10E-01 | 8.10E-01 | 3.10E-01 | 1.98E-02 | 1.00 | 0.71 | 0.01 | 0.00 |
| 2.29 | 3.66 | ENSG00000126860 | EVI2A | Sr | Summer | 0 | 8.18E-03 | 8.00E-02 | 5.00E-02 | 2.60E-01 | 1.00 | 0.27 | 0.35 | 0.02 |
| 2.29 | 3.64 | ENSG00000217416 | ISCA1P1 | Sr | Winter | 0 | 2.00E-01 | 8.00E-02 | 1.90E-01 | 3.54E-03 | 0.53 | 0.37 | -6.07 | -0.51 |
| 2.24 | 3.55 | ENSG00000158966 | CACHD1 | T | Winter | 0 | 8.00E-02 | 1.90E-01 | 2.70E-01 | 7.53E-03 | 0.60 | 0.25 | -2.28 | -0.17 |
| 2.24 | 3.54 | ENSG00000218602 | #NA# | Sr | Winter | 0 | 6.10E-01 | 1.90E-01 | 1.30E-01 | 4.46E-04 | 0.46 | 0.20 | 1.10 | 0.10 |
| 2.23 | 3.53 | ENSG00000216477 | #NA# | T | Winter | 0 | 8.20E-01 | 6.00E-02 | 5.40E-01 | 4.24E-03 | 0.47 | 0.27 | -1.10 | -0.07 |
| 2.22 | 3.52 | ENSG00000162599 | NFIA | Pr | Winter | 0 | 3.10E-01 | 7.70E-01 | 9.20E-01 | 4.99E-03 | 0.49 | 0.09 | 1.39 | 0.09 |
| 2.20 | 3.46 | ENSG00000130695 | CEP85 | Pr | Summer | 0 | 1.00E+00 | 6.10E-01 | 3.90E-01 | 5.83E-04 | 0.55 | 0.18 | -10.78 | -0.67 |
| 2.20 | 3.46 | ENSG00000163092 | XIRP2 | Sr | Summer | 0 | 2.78E-02 | 1.24E-02 | 9.30E-01 | 9.00E-02 | 0.42 | 0.05 | -5.16 | -0.21 |
| 2.17 | 3.42 | ENSG00000152620 | NADKD1 | Sr | Winter | 0 | 4.20E-01 | 5.00E-01 | 2.82E-02 | 1.75E-03 | 0.47 | 0.05 | -1.17 | -0.08 |
| 2.17 | 3.41 | ENSG00000101425 | BPI | T | Winter | 0 | 7.50E-01 | 4.07E-02 | 1.20E-01 | 4.54E-04 | 1.00 | 0.35 | -1.64 | -0.17 |
| 2.17 | 3.41 | ENSG00000209251 | #NA# | Sr | Winter | 0 | 4.20E-02 | 5.70E-01 | 9.70E-01 | 5.54E-03 | 0.56 | 0.17 | -0.94 | -0.06 |
| 2.15 | 3.37 | ENSG00000207637 | MIR595 | Sr | Winter | 0 | 1.80E-01 | 3.60E-01 | 3.09E-02 | 4.34E-03 | 1.00 | 0.85 | -1.69 | -0.07 |
| 2.14 | 3.34 | ENSG00000216553 | #NA# | Pr | Summer | 0 | 9.50E-01 | 7.30E-01 | 7.00E-01 | 3.00E-06 | 0.43 | 0.55 | -9.76 | -0.57 |
| 2.12 | 3.30 | ENSG00000136504 | KAT7 | Pr | Summer | 0 | 9.90E-01 | 4.30E-01 | 4.20E-01 | 1.90E-03 | 0.46 | 0.82 | 2.41 | 0.21 |
| 2.07 | 3.20 | ENSG00000203666 | EFCAB2 | Sr | Summer | 0 | 4.63E-02 | 6.00E-02 | 4.80E-01 | 4.72E-02 | 0.46 | 0.33 | -6.10 | -0.34 |
| 2.05 | 3.18 | ENSG00000218774 | #NA# | Pr | Winter | 0 | 7.00E-01 | 2.70E-01 | 5.00E-01 | 1.00E-01 | 1.00 | 0.05 | -8.49 | -0.45 |
| 2.03 | 3.13 | ENSG00000201405 | #NA# | Sr | Winter | 0 | 5.60E-01 | 1.80E-01 | 1.52E-02 | 5.41E-03 | 0.48 | 0.89 | 1.85 | 0.14 |
| 1.99 | 3.05 | ENSG00000142669 | SH3BGRL3 | Pr | Summer | 0 | 9.90E-01 | 5.20E-01 | 3.90E-01 | 3.74E-04 | 0.52 | 0.26 | -11.70 | -0.61 |
| NA | NA | ENSG00000000938 | FGR | Sr | Winter | 1 | 5.00E-02 | 3.20E-01 | 5.00E-02 | 3.79E-04 | 0.55 | 0.82 | -9.95 | -0.50 |
| NA | NA | ENSG00000211030 | #NA# | T | Winter | 1 | 5.19E-03 | 1.70E-01 | 7.85E-03 | 2.31E-03 | 0.45 | 0.15 | -7.03 | -0.29 |
| NA | NA | ENSG00000215864 | NBPF7 | Pr | Summer | 1 | 4.00E-01 | 9.70E-01 | 1.80E-01 | 6.00E-05 | 0.52 | 0.15 | 4.51 | 0.30 |
| NA | NA | ENSG00000219131 | #NA# | Pr | Summer | 1 | 4.10E-01 | 6.30E-01 | 2.60E-01 | 5.50E-05 | 0.46 | 0.22 | 6.74 | 0.44 |
| NA | NA | ENSG00000213062 | #NA# | T | Summer | 1 | 2.56E-02 | 1.30E-01 | 1.50E-01 | 4.28E-04 | 0.55 | 0.18 | -10.79 | -0.64 |
| NA | NA | ENSG00000198734 | F5 | T | Summer | 1 | 8.46E-03 | 6.98E-03 | 3.80E-01 | 2.11E-03 | 1.00 | 0.11 | -6.57 | -0.50 |
| NA | NA | ENSG00000220782 | #NA# | T | Winter | 1 | 1.68E-02 | 2.16E-02 | 1.50E-01 | 1.50E-04 | 1.00 | 0.12 | -4.25 | -0.42 |
| NA | NA | ENSG00000212094 | #NA# | T | Summer | 1 | 6.93E-03 | 5.00E-02 | 7.00E-02 | 2.30E-03 | 0.48 | 0.30 | -4.39 | -0.42 |
| NA | NA | ENSG00000219780 | #NA# | Sr | Winter | 1 | 5.00E-02 | 1.10E-01 | 4.10E-02 | 5.46E-04 | 0.54 | 0.66 | -20.30 | -0.72 |
| NA | NA | ENSG00000197454 | OR2L5 | T | Winter | 0 | 2.57E-03 | 9.80E-01 | 7.80E-01 | 1.90E-02 | 0.48 | 0.22 | -5.95 | -0.35 |
| NA | NA | ENSG00000205795 | CYS1 | T | Winter | 1 | 8.59E-03 | 7.30E-01 | 1.90E-02 | 1.64E-04 | 0.57 | 0.30 | -5.99 | -0.60 |
| NA | NA | ENSG00000219820 | #NA# | T | Winter | 1 | 1.50E-01 | 3.30E-01 | 8.00E-02 | 2.24E-03 | 0.49 | 0.08 | -5.67 | -0.33 |
| NA | NA | ENSG00000171150 | SOCS5 | Sr | Winter | 1 | 1.05E-02 | 5.50E-01 | 3.14E-02 | 2.88E-03 | 0.57 | 0.09 | -11.18 | -0.55 |
| NA | NA | ENSG00000179843 | #NA# | Sr | Winter | 1 | 1.18E-02 | 2.26E-02 | 7.84E-03 | 6.20E-05 | 0.50 | 0.67 | -8.09 | -0.31 |
| NA | NA | ENSG00000179843 | #NA# | T | Winter | 1 | 2.37E-02 | 9.00E-02 | 1.60E-01 | 1.59E-03 | 1.00 | 0.29 | -8.09 | -0.31 |
| NA | NA | ENSG00000208374 | #NA# | Sr | Winter | 1 | 7.00E-02 | 1.06E-02 | 3.70E-02 | 5.60E-05 | 0.59 | 0.67 | -10.54 | -0.40 |
| NA | NA | ENSG00000171135 | JAGN1 | Sr | Winter | 1 | 8.00E-02 | 8.00E-01 | 1.08E-02 | 8.00E-06 | 0.45 | 0.17 | -11.91 | -0.56 |
| NA | NA | ENSG00000163701 | IL17RE | Sr | Winter | 1 | 1.60E-01 | 7.50E-01 | 2.04E-02 | 7.00E-06 | 0.47 | 0.20 | -12.34 | -0.53 |
| NA | NA | ENSG00000163810 | TGM4 | Sr | Winter | 1 | 3.21E-02 | 1.80E-01 | 1.98E-02 | 3.20E-05 | 0.47 | 0.68 | -1.53 | -0.10 |
| NA | NA | ENSG00000114302 | PRKAR2A | Sr | Winter | 1 | 9.00E-02 | 8.10E-01 | 3.50E-02 | 2.94E-04 | 0.52 | 0.23 | -6.94 | -0.30 |
| NA | NA | ENSG00000211273 | #NA# | T | Winter | 1 | 1.04E-03 | 2.80E-01 | 1.72E-02 | 1.37E-02 | 0.39 | 0.05 | -14.53 | -0.57 |
| NA | NA | ENSG00000201314 | #NA# | Sr | Winter | 1 | 1.67E-02 | 5.20E-01 | 1.02E-02 | 1.51E-03 | 0.58 | 0.62 | -5.39 | -0.51 |
| NA | NA | ENSG00000211394 | #NA# | Pr | Summer | 1 | 2.10E-01 | 3.10E-01 | 6.00E-02 | 4.03E-03 | 0.48 | 0.03 | -9.37 | -0.34 |
| NA | NA | ENSG00000210787 | #NA# | Sr | Winter | 1 | 2.05E-04 | 5.70E-01 | 1.40E-01 | 3.00E-03 | 0.56 | 0.70 | -3.96 | -0.32 |
| NA | NA | ENSG00000095970 | TREM2 | T | Winter | 1 | 2.70E-01 | 2.00E-01 | 6.25E-03 | 4.21E-04 | 0.51 | 0.33 | 1.30 | 0.08 |
| NA | NA | ENSG00000096080 | MRPS18A | Sr | Winter | 1 | 4.43E-04 | 6.00E-02 | 3.52E-02 | 9.34E-04 | 1.00 | 0.37 | -7.30 | -0.35 |
| NA | NA | ENSG00000164430 | MB21D1 | Pr | Summer | 1 | 3.40E-01 | 6.90E-01 | 7.00E-02 | 1.22E-04 | 0.54 | 0.08 | -9.76 | -0.58 |
| NA | NA | ENSG00000203863 | #NA# | Pr | Summer | 1 | 3.20E-01 | 1.50E-01 | 1.20E-01 | 1.21E-03 | 0.46 | 0.04 | -6.68 | -0.38 |
| NA | NA | ENSG00000146263 | MMS22L | Sr | Winter | 1 | 2.97E-04 | 4.80E-01 | 1.12E-02 | 2.67E-02 | 0.41 | 0.60 | -15.18 | -0.71 |
| NA | NA | ENSG00000207461 | #NA# | Sr | Winter | 1 | 4.83E-03 | 2.30E-01 | 6.00E-02 | 1.83E-02 | 0.47 | 0.16 | -5.96 | -0.52 |
| NA | NA | ENSG00000219302 | #NA# | Pr | Summer | 1 | 4.00E-02 | 8.60E-01 | 2.40E-01 | 4.20E-05 | 0.54 | 0.04 | -7.69 | -0.57 |
| NA | NA | ENSG00000200058 | #NA# | Pr | Summer | 1 | 8.70E-01 | 9.30E-01 | 1.57E-02 | 1.90E-05 | 0.51 | 0.19 | 1.22 | 0.13 |
| NA | NA | ENSG00000152822 | GRM1 | Pr | Summer | 1 | 1.20E-01 | 1.00E-01 | 1.72E-02 | 1.53E-04 | 0.46 | 0.19 | -0.87 | -0.06 |
| NA | NA | ENSG00000105792 | C7orf63 | Pr | Summer | 1 | 4.50E-01 | 8.30E-01 | 2.10E-01 | 1.80E-05 | 0.42 | 0.10 | -3.26 | -0.26 |
| NA | NA | ENSG00000208283 | #NA# | Pr | Summer | 1 | 4.00E-01 | 3.60E-01 | 5.00E-02 | 1.50E-03 | 0.51 | 0.05 | -10.89 | -0.42 |
| NA | NA | ENSG00000166228 | PCBD1 | Pr | Summer | 1 | 4.20E-01 | 3.20E-01 | 8.07E-03 | 1.52E-04 | 0.52 | 0.16 | -6.51 | -0.50 |
| NA | NA | ENSG00000214688 | C10orf105 | T | Winter | 0 | 7.00E-02 | 9.10E-01 | 7.90E-01 | 1.00E-06 | 0.51 | 0.27 | -1.43 | -0.19 |
| NA | NA | ENSG00000213410 | #NA# | Sr | Winter | 1 | 2.55E-03 | 5.80E-01 | 9.40E-01 | 4.29E-04 | 0.46 | 0.25 | 8.21 | 0.31 |
| NA | NA | ENSG00000172893 | DHCR7 | Sr | Winter | 1 | 3.80E-01 | 4.80E-01 | 8.98E-03 | 9.23E-04 | 0.50 | 0.09 | 3.31 | 0.19 |
| NA | NA | ENSG00000137710 | RDX | Pr | Summer | 0 | 6.50E-01 | 1.00E-01 | 1.10E-01 | 8.00E-03 | 0.52 | 0.38 | -23.33 | -0.74 |
| NA | NA | ENSG00000167548 | MLL2 | Sr | Summer | 1 | 2.16E-03 | 3.82E-02 | 3.00E-01 | 1.15E-02 | 0.45 | 0.19 | -4.13 | -0.33 |
| NA | NA | ENSG00000167550 | RHEBL1 | Sr | Summer | 1 | 4.66E-03 | 9.00E-02 | 5.70E-01 | 7.01E-03 | 0.46 | 0.28 | -5.35 | -0.32 |
| NA | NA | ENSG00000123307 | NEUROD4 | T | Winter | 1 | 1.33E-03 | 2.00E-01 | 4.10E-01 | 1.03E-03 | 0.50 | 0.68 | -2.85 | -0.27 |
| NA | NA | ENSG00000220367 | #NA# | Pr | Summer | 1 | 9.10E-01 | 5.10E-01 | 1.10E-01 | 3.40E-05 | 0.55 | 0.08 | -5.15 | -0.29 |
| NA | NA | ENSG00000216569 | #NA# | Sr | Winter | 1 | 8.00E-02 | 1.50E-01 | 3.40E-02 | 5.13E-04 | 0.45 | 0.37 | -13.27 | -0.77 |
| NA | NA | ENSG00000005810 | MYCBP2 | Sr | Winter | 1 | 5.00E-02 | 4.50E-01 | 5.00E-02 | 2.95E-04 | 0.43 | 0.27 | -6.79 | -0.28 |
| NA | NA | ENSG00000212335 | #NA# | T | Winter | 1 | 1.23E-03 | 2.81E-02 | 6.00E-02 | 8.76E-03 | 1.00 | 0.08 | -9.53 | -0.73 |
| NA | NA | ENSG00000129480 | C14orf126 | rH | Winter | 1 | 1.60E-01 | 4.55E-02 | 2.30E-01 | 1.60E-05 | 0.59 | 0.35 | -2.00 | -0.12 |
| NA | NA | ENSG00000209604 | #NA# | Sr | Winter | 0 | 3.90E-01 | 5.30E-01 | 4.89E-03 | 1.16E-04 | 0.47 | 0.44 | -7.90 | -0.60 |
| NA | NA | ENSG00000201358 | #NA# | Pr | Summer | 1 | 1.20E-01 | 5.60E-01 | 5.00E-02 | 4.51E-03 | 0.53 | 0.05 | -10.91 | -0.73 |
| NA | NA | ENSG00000053770 | MUDENG | Sr | Winter | 0 | 1.86E-02 | 3.50E-01 | 1.25E-02 | 3.79E-03 | 0.47 | 0.62 | -3.41 | -0.27 |
| NA | NA | ENSG00000165555 | NOXRED1 | Sr | Winter | 1 | 9.00E-02 | 1.10E-01 | 3.29E-02 | 1.58E-03 | 0.54 | 0.54 | -8.64 | -0.39 |
| NA | NA | ENSG00000126214 | KLC1 | Sr | Winter | 0 | 1.60E-01 | 8.00E-02 | 3.50E-02 | 9.00E-02 | 0.55 | 0.06 | 5.20 | 0.33 |
| NA | NA | ENSG00000166173 | LARP6 | Pr | Winter | 1 | 1.50E-01 | 8.00E-02 | 1.40E-02 | 1.13E-02 | 0.53 | 0.24 | -10.38 | -0.55 |
| NA | NA | ENSG00000158486 | DNAH3 | T | Winter | 1 | 1.00E-01 | 4.10E-01 | 1.30E-01 | 7.80E-05 | 0.45 | 0.12 | 3.65 | 0.24 |
| NA | NA | ENSG00000213704 | EEF1A1P15 | T | Winter | 1 | 5.00E-02 | 2.60E-01 | 1.37E-02 | 1.41E-03 | 1.00 | 0.12 | 1.32 | 0.10 |
| NA | NA | ENSG00000177548 | RABEP2 | Sr | Summer | 1 | 1.82E-03 | 4.55E-02 | 7.00E-01 | 3.01E-03 | 0.48 | 0.42 | -15.68 | -0.48 |
| NA | NA | ENSG00000166152 | C16orf78 | Sr | Winter | 1 | 6.20E-01 | 3.91E-02 | 1.45E-02 | 5.40E-05 | 0.49 | 0.74 | -6.27 | -0.37 |
| NA | NA | ENSG00000214470 | #NA# | Sr | Winter | 1 | 9.14E-04 | 1.50E-01 | 2.69E-02 | 9.86E-03 | 0.53 | 0.80 | -13.20 | -0.78 |
| NA | NA | ENSG00000222190 | MIR1910 | Sr | Winter | 1 | 3.90E-01 | 3.20E-01 | 5.35E-03 | 3.50E-05 | 0.49 | 0.43 | -8.91 | -0.57 |
| NA | NA | ENSG00000181464 | #NA# | T | Winter | 1 | 7.33E-03 | 6.20E-01 | 8.83E-03 | 8.50E-04 | 0.60 | 0.51 | -6.09 | -0.36 |
| NA | NA | ENSG00000187451 | #NA# | T | Winter | 1 | 9.04E-03 | 5.80E-01 | 3.68E-02 | 1.36E-04 | 0.45 | 0.88 | -7.86 | -0.32 |
| NA | NA | ENSG00000176358 | TAC4 | Pr | Summer | 0 | 8.30E-01 | 7.40E-01 | 2.70E-01 | 6.25E-04 | 0.51 | 0.66 | 9.96 | 0.54 |
| NA | NA | ENSG00000200875 | #NA# | rH | Summer | 1 | 1.83E-03 | 2.97E-03 | 1.30E-01 | 8.00E-02 | 0.46 | 0.10 | -0.24 | -0.02 |
| NA | NA | ENSG00000212067 | #NA# | Sr | Winter | 1 | 1.84E-04 | 1.60E-01 | 2.70E-01 | 5.50E-05 | 0.47 | 0.33 | -2.79 | -0.21 |
| NA | NA | ENSG00000200132 | #NA# | Pr | Summer | 0 | 9.90E-01 | 3.00E-01 | 1.40E-01 | 2.75E-03 | 1.00 | 0.17 | 8.44 | 0.38 |
| NA | NA | ENSG00000053501 | USE1 | T | Winter | 1 | 1.20E-01 | 8.00E-02 | 2.59E-02 | 3.00E-03 | 0.47 | 0.09 | -10.80 | -0.63 |
| NA | NA | ENSG00000099330 | OCEL1 | T | Winter | 1 | 7.00E-02 | 2.80E-01 | 4.42E-02 | 6.05E-03 | 0.50 | 0.06 | -7.53 | -0.61 |
| NA | NA | ENSG00000198153 | #NA# | T | Winter | 1 | 8.18E-03 | 2.09E-02 | 2.16E-02 | 1.35E-02 | 0.45 | 0.25 | -0.97 | -0.05 |
| NA | NA | ENSG00000205243 | #NA# | Sr | Winter | 1 | 3.80E-03 | 1.95E-02 | 1.20E-01 | 2.91E-04 | 1.00 | 0.03 | -1.05 | -0.09 |
| NA | NA | ENSG00000090013 | BLVRB | T | Winter | 1 | 8.00E-02 | 3.90E-01 | 4.30E-01 | 1.45E-04 | 0.37 | 0.05 | -12.71 | -0.63 |
| NA | NA | ENSG00000149346 | C20orf94 | T | Winter | 1 | 2.34E-02 | 3.80E-01 | 3.20E-01 | 2.75E-04 | 0.42 | 0.05 | -4.15 | -0.30 |
| NA | NA | ENSG00000209723 | #NA# | Pr | Summer | 1 | 3.66E-03 | 1.20E-01 | 8.00E-02 | 2.19E-03 | 0.57 | 0.22 | 0.64 | 0.07 |
| NA | NA | ENSG00000196756 | #NA# | rH | Summer | 0 | 4.24E-02 | 3.62E-02 | 2.20E-01 | 8.00E-02 | 0.45 | 0.03 | -11.96 | -0.56 |
| NA | NA | ENSG00000160220 | #NA# | T | Winter | 1 | 1.63E-02 | 2.40E-01 | 7.00E-02 | 1.42E-03 | 0.52 | 0.36 | 5.29 | 0.22 |
| NA | NA | ENSG00000100362 | PVALB | Sr | Winter | 1 | 7.40E-03 | 4.30E-01 | 1.50E-01 | 1.34E-03 | 0.48 | 0.28 | -0.02 | 0.00 |
| NA | NA | ENSG00000206813 | #NA# | Sr | Winter | 0 | 1.70E-01 | 7.00E-02 | 9.00E-02 | 7.14E-03 | 0.54 | 0.85 | -6.66 | -0.56 |
